# Supplementary figures and images for: A top-down approach of sources and non-photosynthetic sinks of carbonyl sulfide from atmospheric measurements over multiple years in the Paris region (France)
Source: PLoS One. 2020 Feb 10;15(2):e0228419. doi: 10.1371/journal.pone.0228419 (PMC7010246; doi:10.1371/journal.pone.0228419)

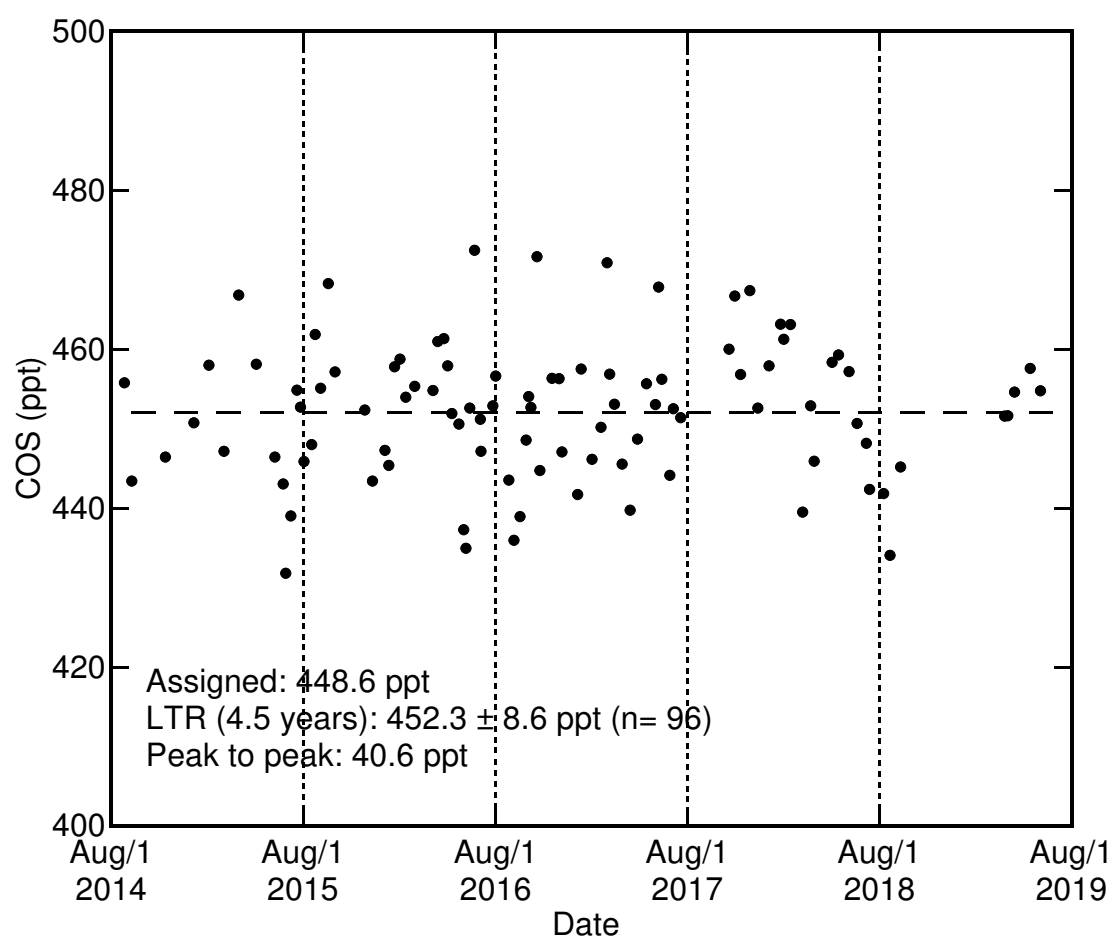

Supplement: S1 Fig — The air compressed cylinder, prepared and certified by NOAA-ESRL, was analyzed by gas chromatography with PFPD detection. The GC was calibrated using a calibration gas purchased from Air Products. The LTR here expressed as the SD of the average of 96 determinations over 4.5 years is 8.6 ppt. From October 2018 to March 2019 the cylinder was not used to increase its service life. (PDF) [file pone.0228419.s001.pdf]

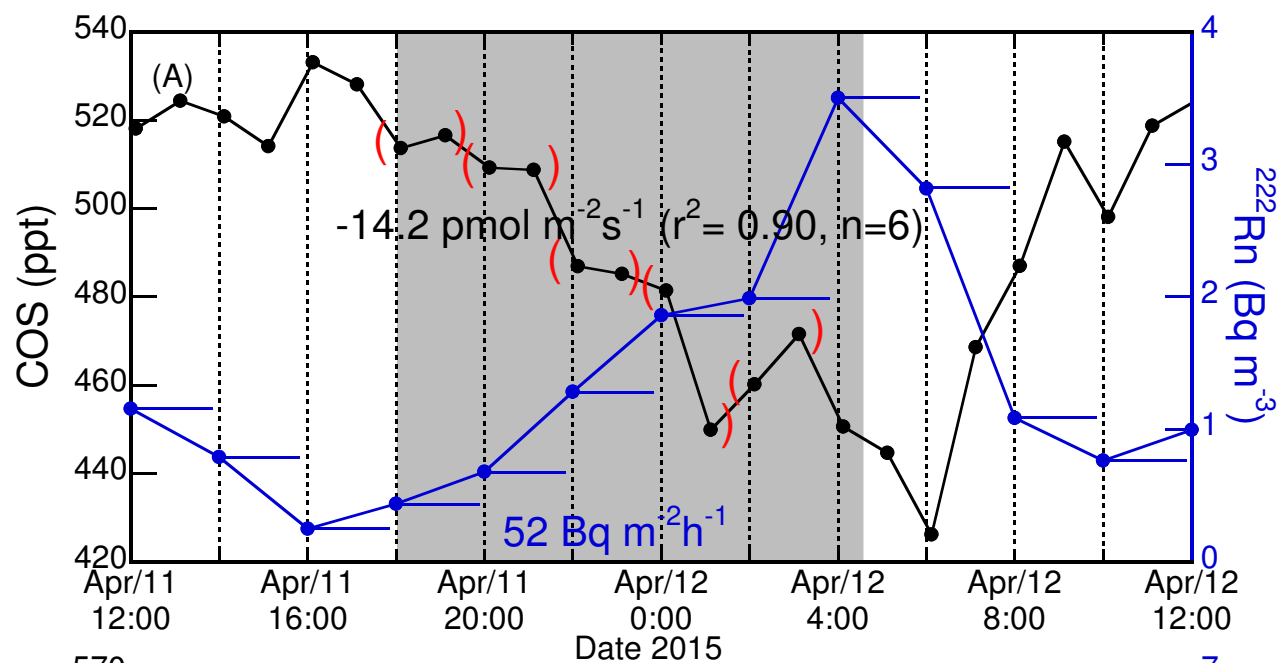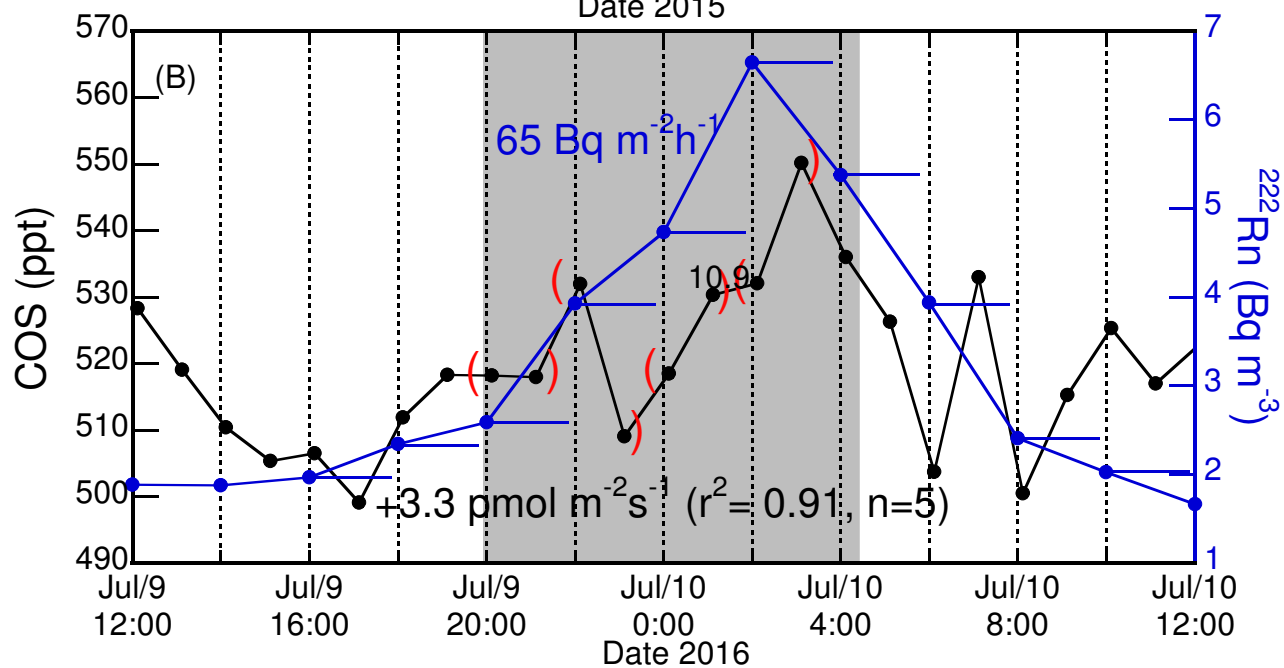

Supplement: S2 Fig — The night length explains why the grey band is narrower in summer than in spring. Drier conditions explain why 222Rn exhalation rates from soil are higher in summer than in spring. The blue horizontal bars indicate that 222Rn is measured in two-hour increments and that corresponding data are reported at the beginning of the two-hour interval. Data between red brackets were averaged and the slope of linear regression during night-time inversion was calculated (n = 6 in spring, n = 5 in summer). Time is UTC. (PDF) [file pone.0228419.s002.pdf]

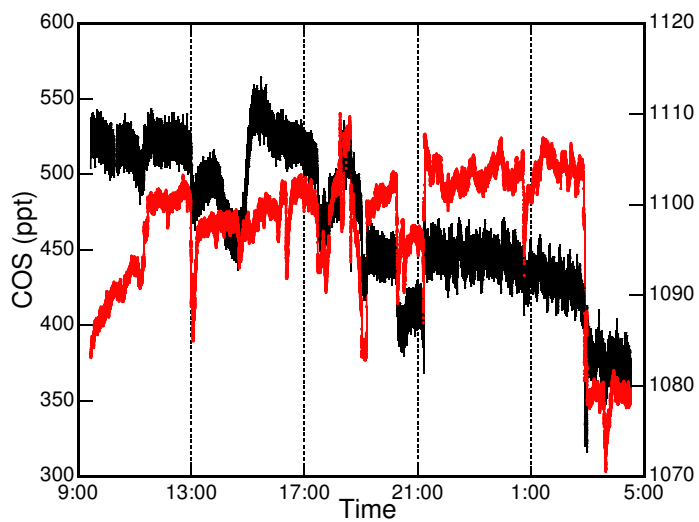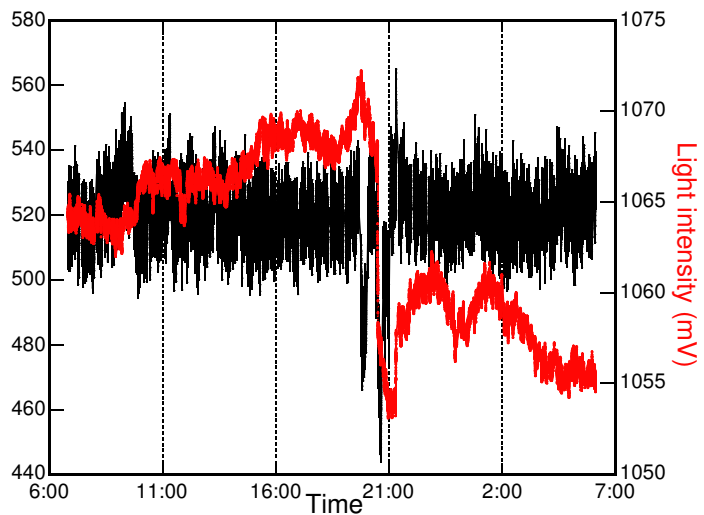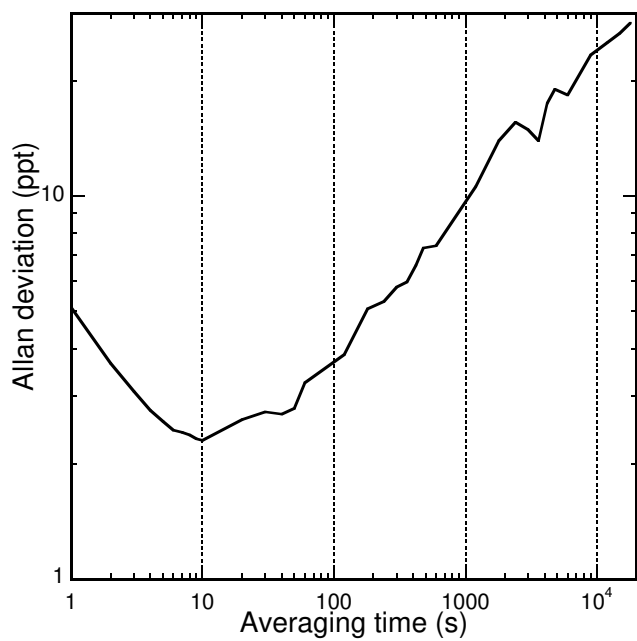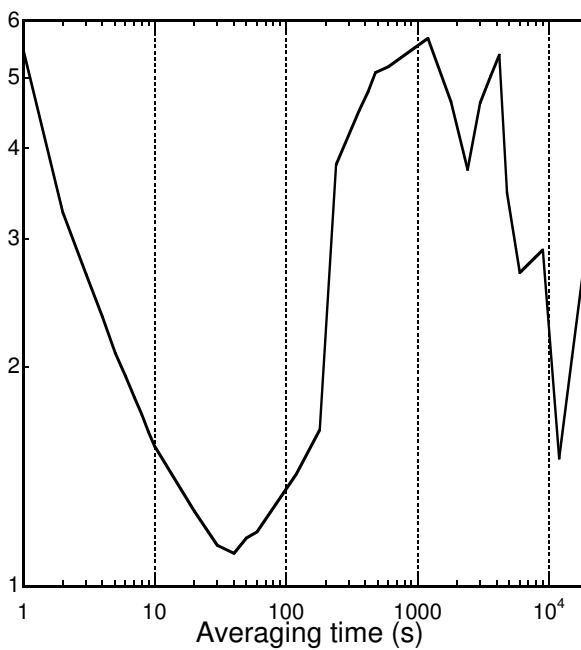

Supplement: S3 Fig — CMR was assessed using a single target gas tank filled with dry natural air measured continuously over time periods of at least 19 h (upper panels showing the temporal variations in COS mixing ratio and laser light intensity), without (left column) and with (right column) “zero” air spectrum measurements. In the latter case, high-purity nitrogen is passed through the cell every 20 minutes for 1 minute at 500 ml/min. The lower panels present the respective Allan deviation from 1 s to 2.104 s averaging time (logarithmic scale). (PDF) [file pone.0228419.s003.pdf]

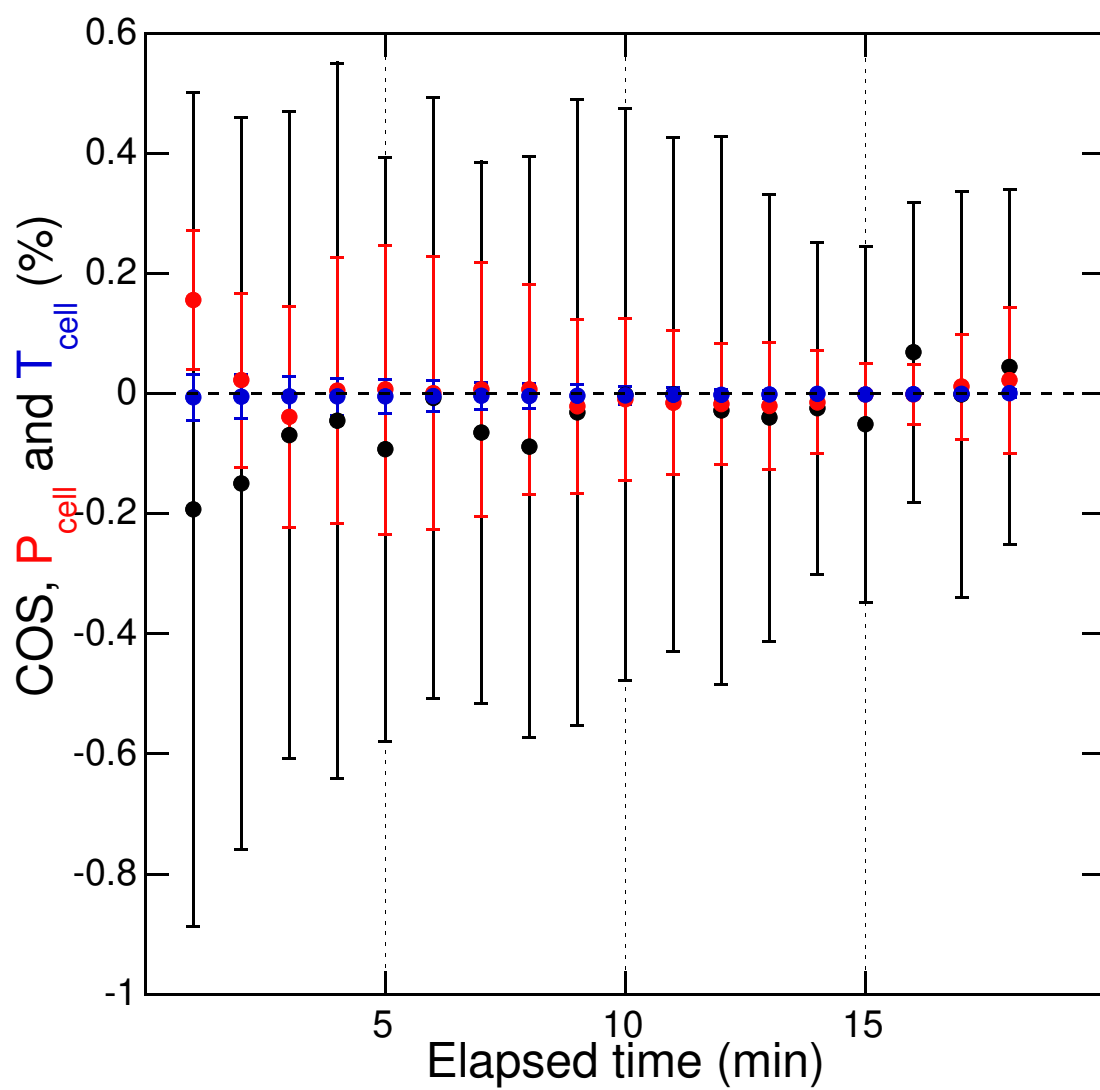

Supplement: S4 Fig — It is the time necessary to reach the final value (calculated over the last 5 min of an analysis) from CMR test with “zero” air spectrum measurements (see S3 Fig, upper right panel). The stabilization time is averaged over 25 injections from a cylinder. Also shown is the stability of the cell pressure and temperature. Cell characteristics: 76 m multi-pass cell, 500 ml of cell volume, 40 Torr of cell pressure. (PDF) [file pone.0228419.s004.pdf]

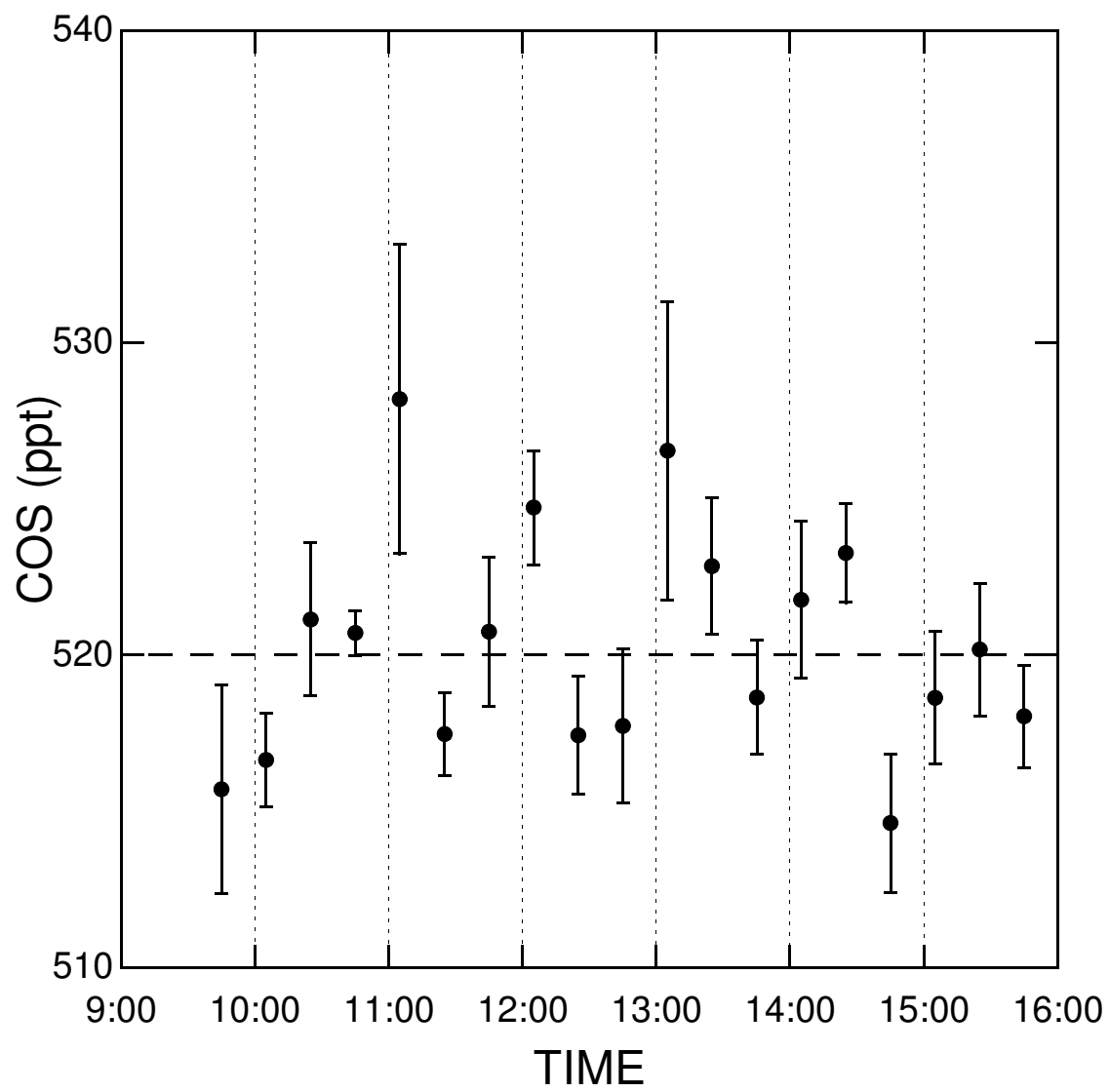

Supplement: S5 Fig — We evaluate the ability of the mini-QCL to always reach the same value for a target gas when alternated with a different sample, high-purity nitrogen injected for 1 min in this case. The target gas was measured 19 times and the first 4 minutes of each injection was discarded to account for stabilization. STR is the SD of 19 consecutive injections. STR = 3.6 ppt. The amplitude peak-to-peak is 13.6 ppt. (PDF) [file pone.0228419.s005.pdf]

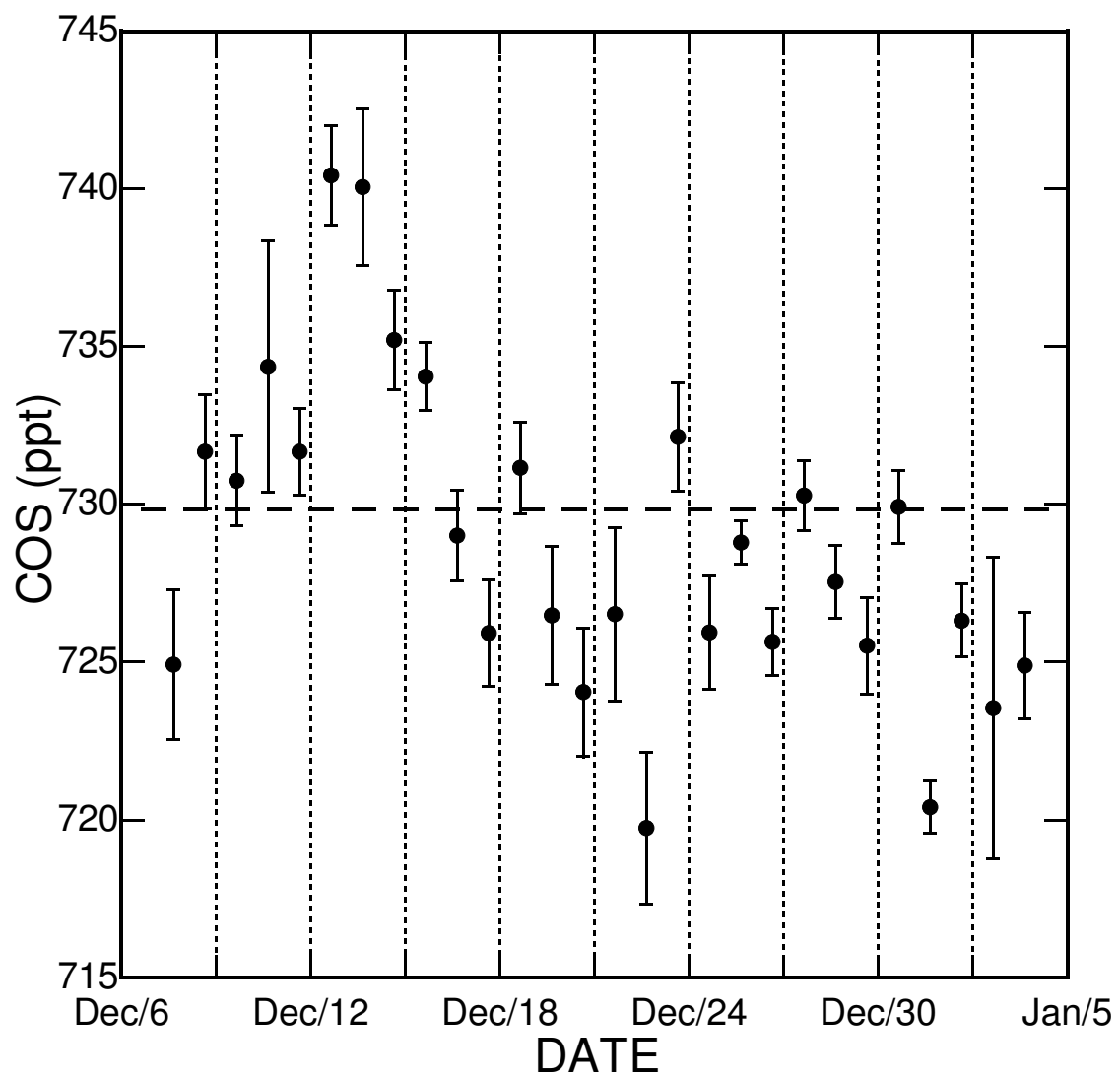

Supplement: S6 Fig — It quantifies the stability of the mini-QCL over about a month. A target gas was measured once a day alternating with ambient air measurements. The last 10 minutes of each 20 min injection were taken into account for averaging. Calibrated data are required for LTR assessment. LTR = 5.1 ppt. The amplitude peak-to-peak is 20.7 ppt. (PDF) [file pone.0228419.s006.pdf]

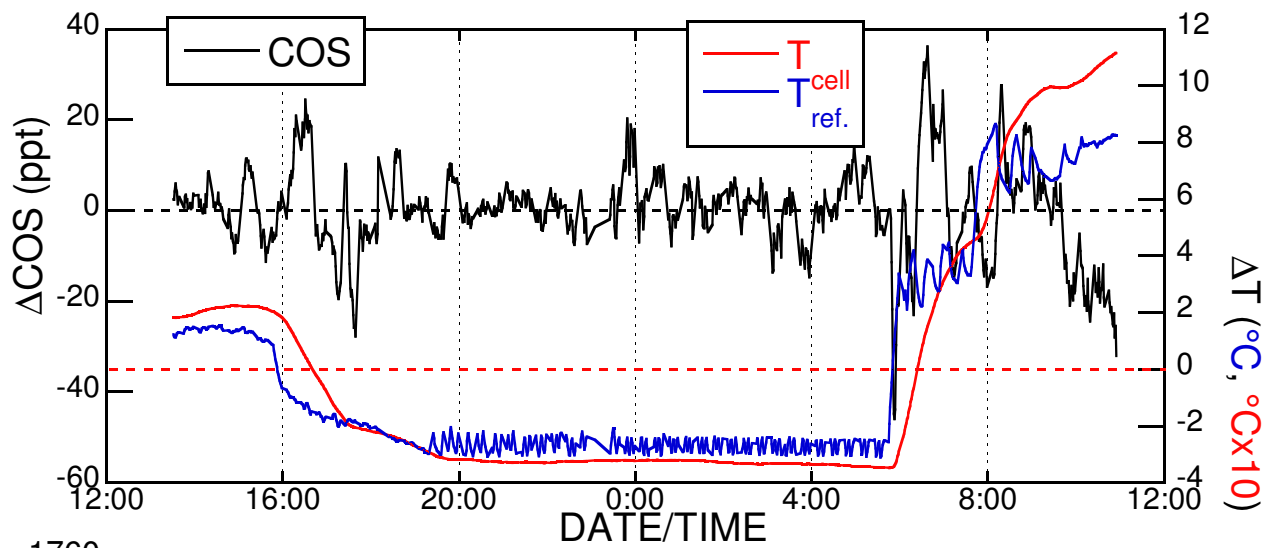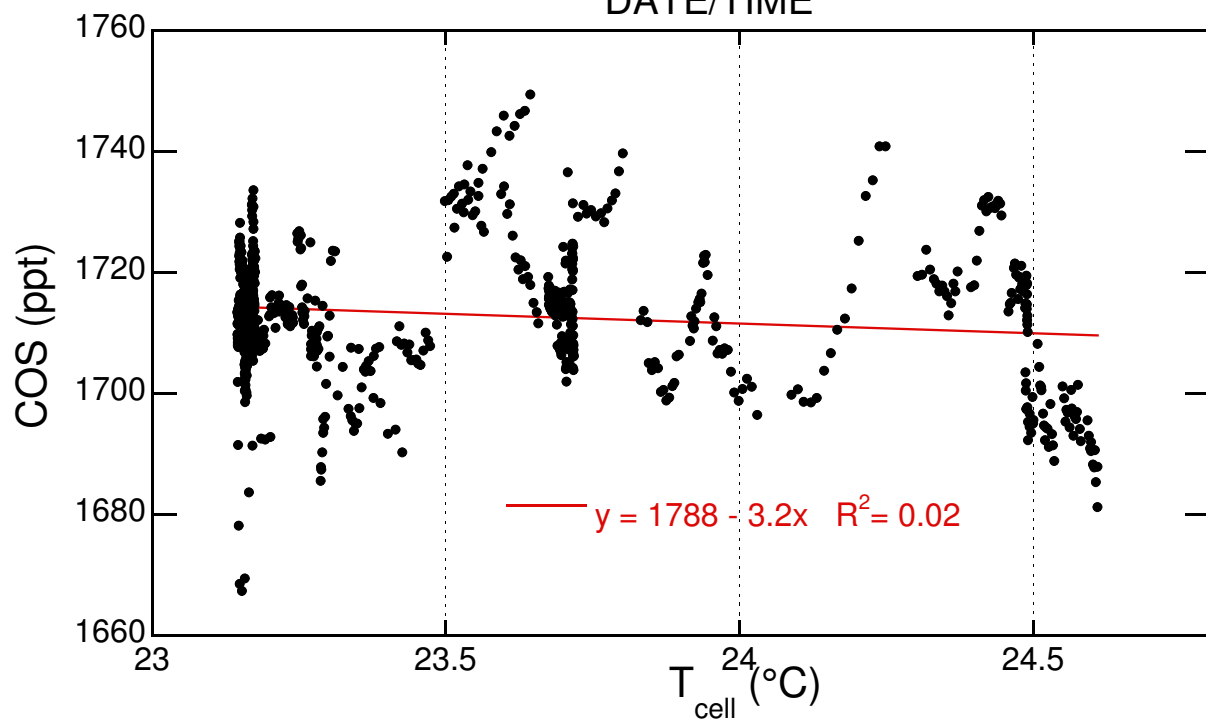

Supplement: S7 Fig — The sensitivity of the mini-QCL to large room temperature variations (20.7–32.5 °C) is assessed. The top panel presents the time series of concentration (black, 1 min averages), the room temperature in the laboratory (blue) and the temperature in the cell (red). Data are expressed as deviations from the daily average. The temperature in the cell was multiplied by 10 to make the variations visible on the same scale as the room temperature (right axis). The concentration of COS is plotted against the cell temperature in the lower panel. The large COS variations (about 80 ppt peak-to-peak) cannot be corrected for changes in Tcell. (PDF) [file pone.0228419.s007.pdf]

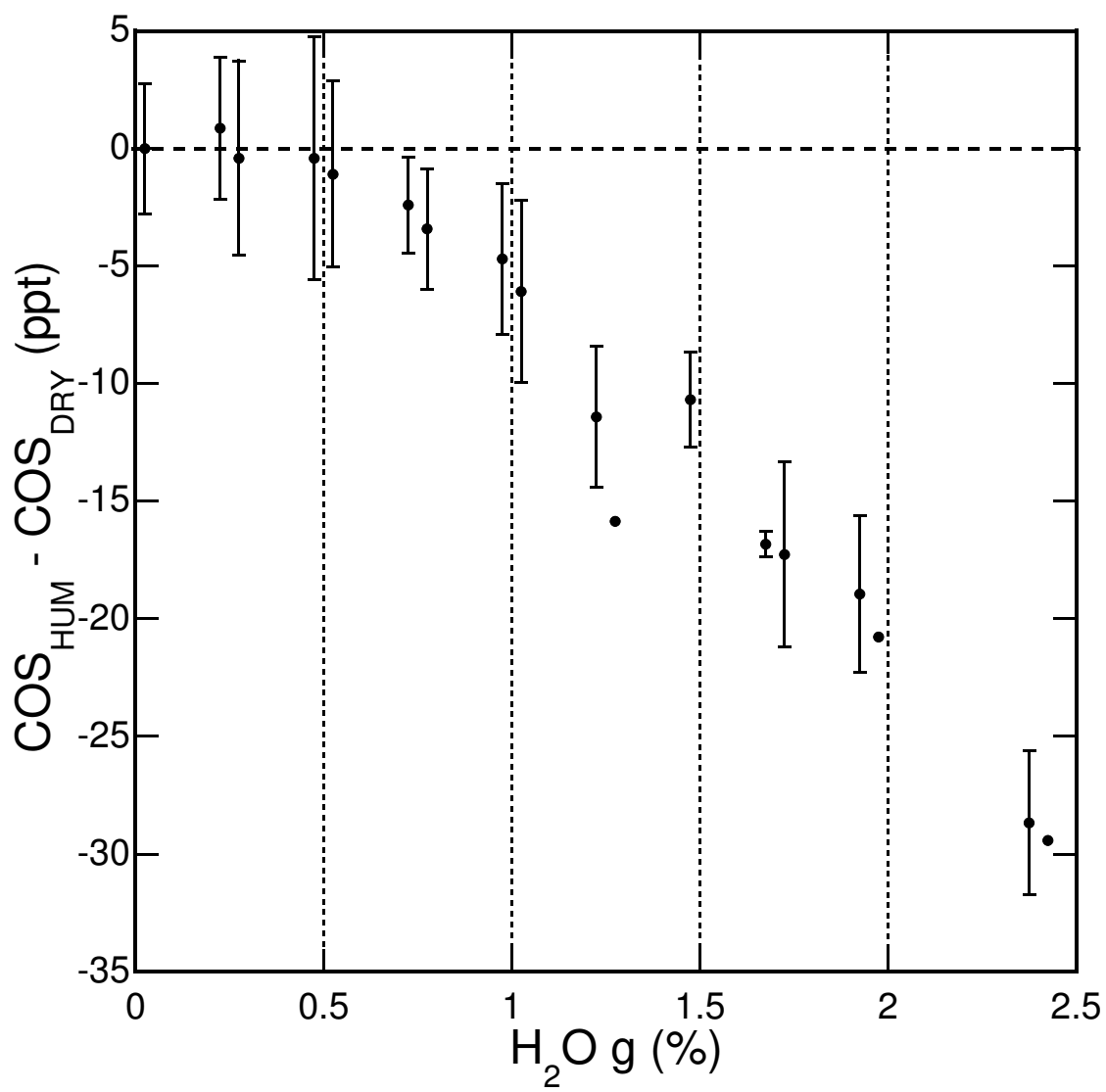

Supplement: S8 Fig — All the data were averaged over 1 min. The humidifying bench is composed of one thermal mass flow controller (F-201CV, Bronkhorst), to regulate the flow of a tank filled with dry natural air, one liquid mass flow controller (Mini Cori-Flow M12, Bronkhorst), to regulate the quantity of Milli-Q water injected in the sample line, and one controlled evaporator mixer (Bronkhorst) to humidify the target gas by evaporating the water at 40 °C while mixing it with the gas. (PDF) [file pone.0228419.s008.pdf]

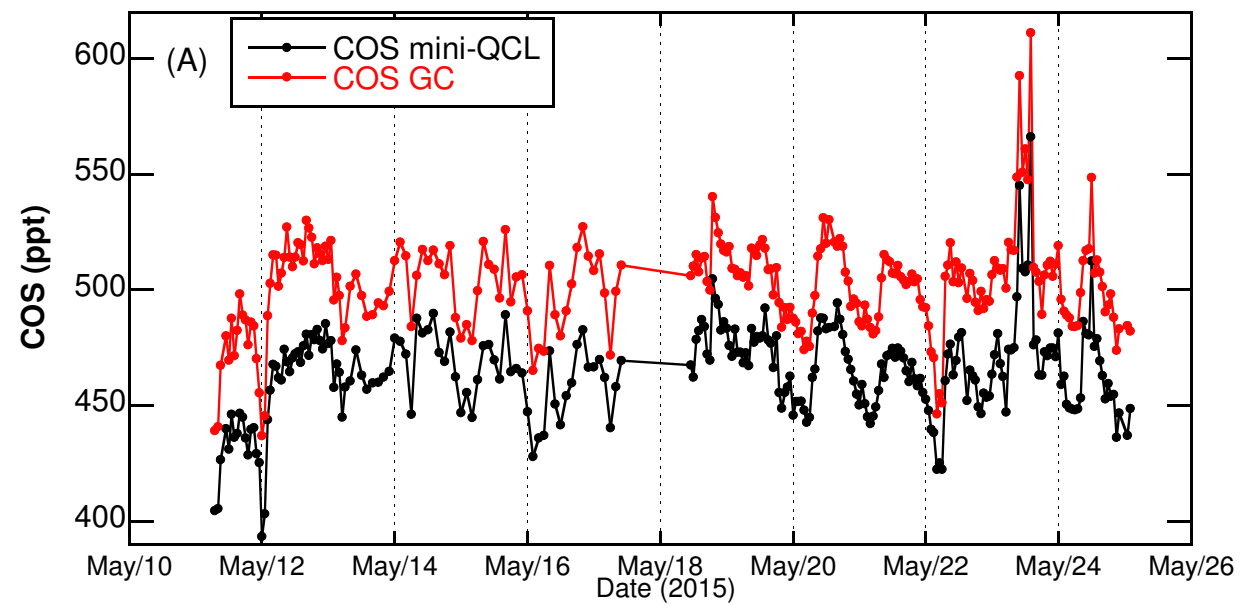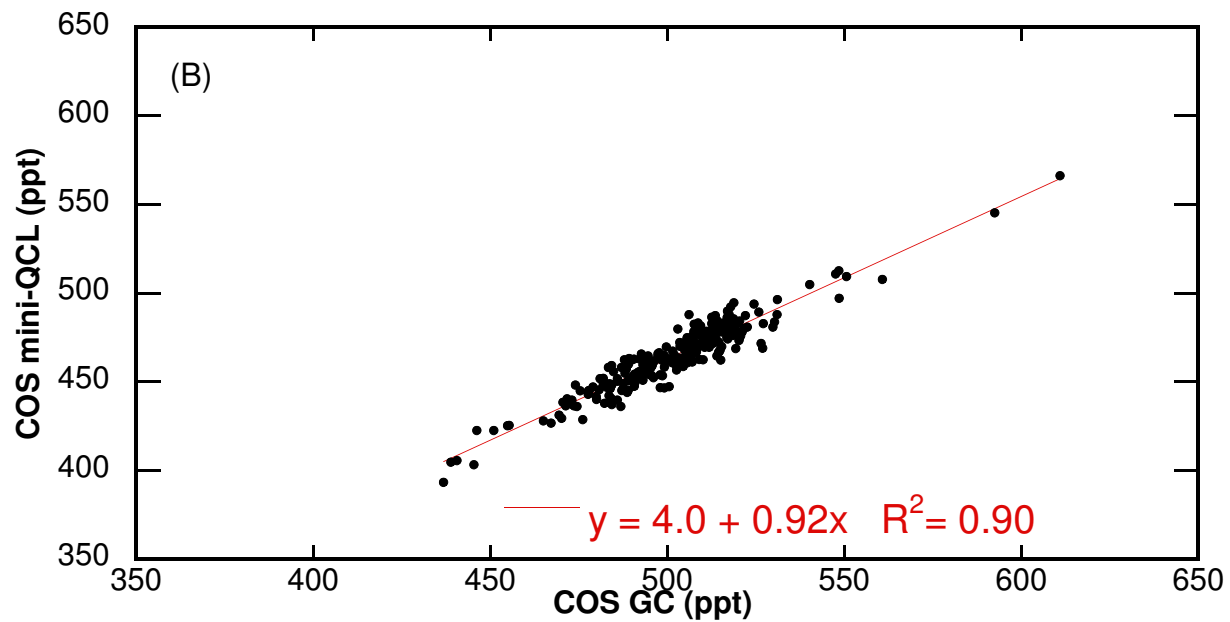

Supplement: S9 Fig — Ambient air measurement comparisons at Gif-sur-Yvette (May 2015). After water correction, the mini-QCL measurements were 5 min averaged to allow for meaningful comparisons with GC discrete measurements. The lower panel shows the linear regression between both datasets. The ordinate (4 ppt) is not significant at the level of 5% and the 95% confidence interval of the slope is 0.88–0.95. (PDF) [file pone.0228419.s009.pdf]

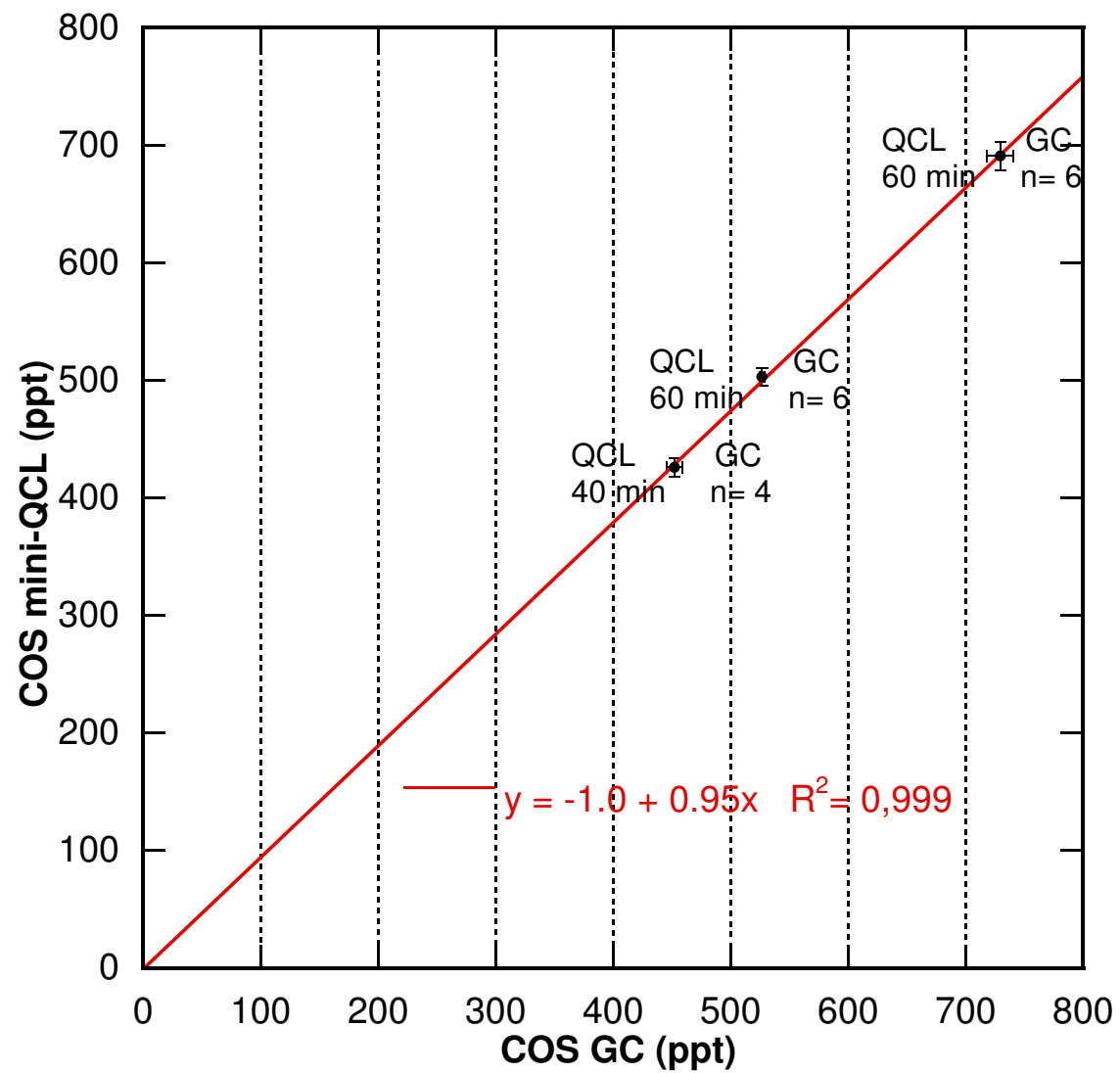

Supplement: S10 Fig — Performed using three air compressed cylinders, only one being Aculife-treated (NOAA-ESRL, 448.6 ± 0.2 ppt GC-MS analysis, 452.3 ± 7.0 ppt GC-PFPD analysis). The COS content of the un-treated aluminum cylinders was 526.7 ± 2.2 ppt and 729.6 ± 11.3 ppt (GC-PFPD analyses). Errors bars are 1 SD of XX min and XY replicates of compressed air measured by mini-QCL and GC, respectively. (PDF) [file pone.0228419.s010.pdf]

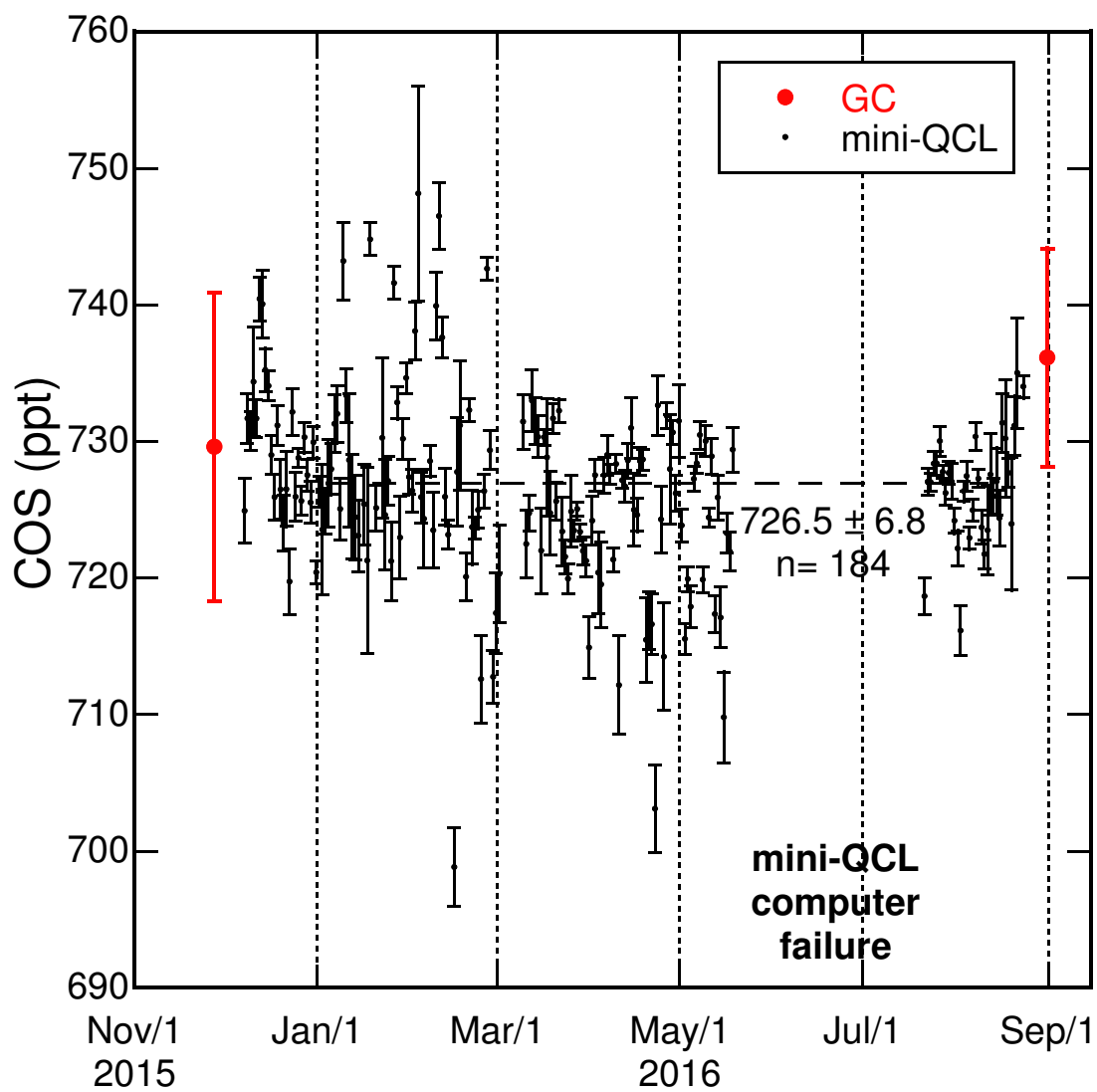

Supplement: S11 Fig — A target gas was measured once a day alternating with ambient air and calibration gas measurements (every 10 hours in the latter case). The last 10 minutes of each 20 min injection were taken into account for averaging. Data were calibrated as described in text (i.e. [COS]target final = 1.0547[COS]dry + B, with B = [COS]certified− 1.0547[COS]raw and [COS]certified = 526.7 ± 2.2 ppt (dry gas)). The target gas was analyzed by GC-PFPD at the beginning and at the end of the survey (red dots), and in March 2017 too (744.9 ± 7.6 ppt). This demonstrates that COS can be conservative for at least 16 months also in un-treated aluminum cylinders. The failure of the mini-QCL computer is responsible for the data gap in June-July 2016. (PDF) [file pone.0228419.s011.pdf]

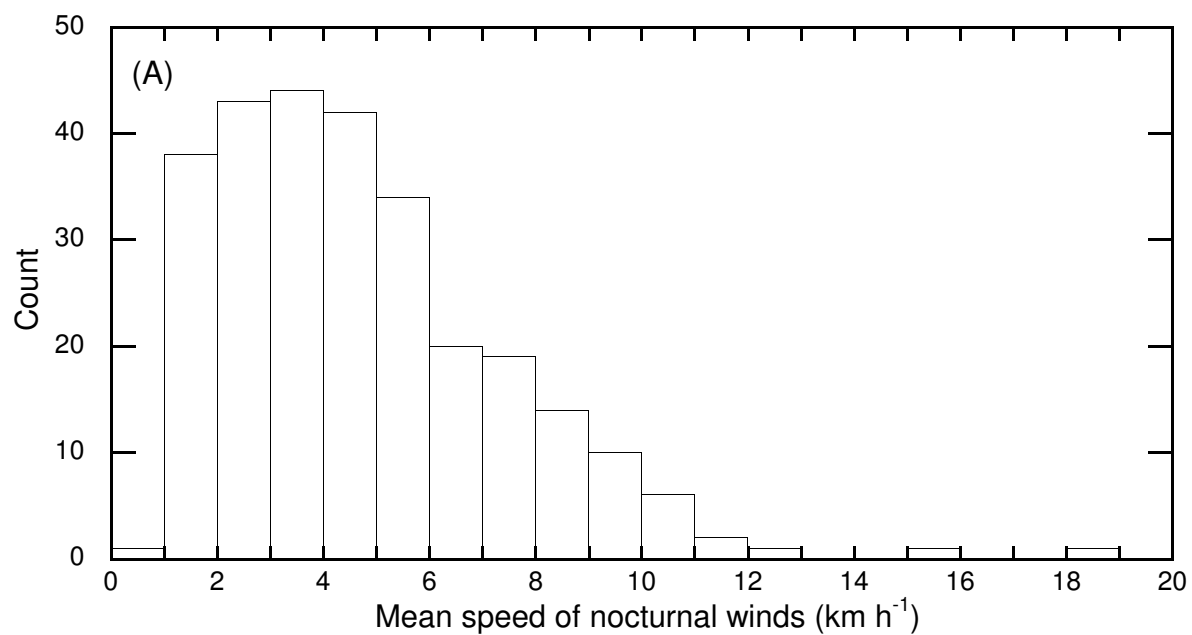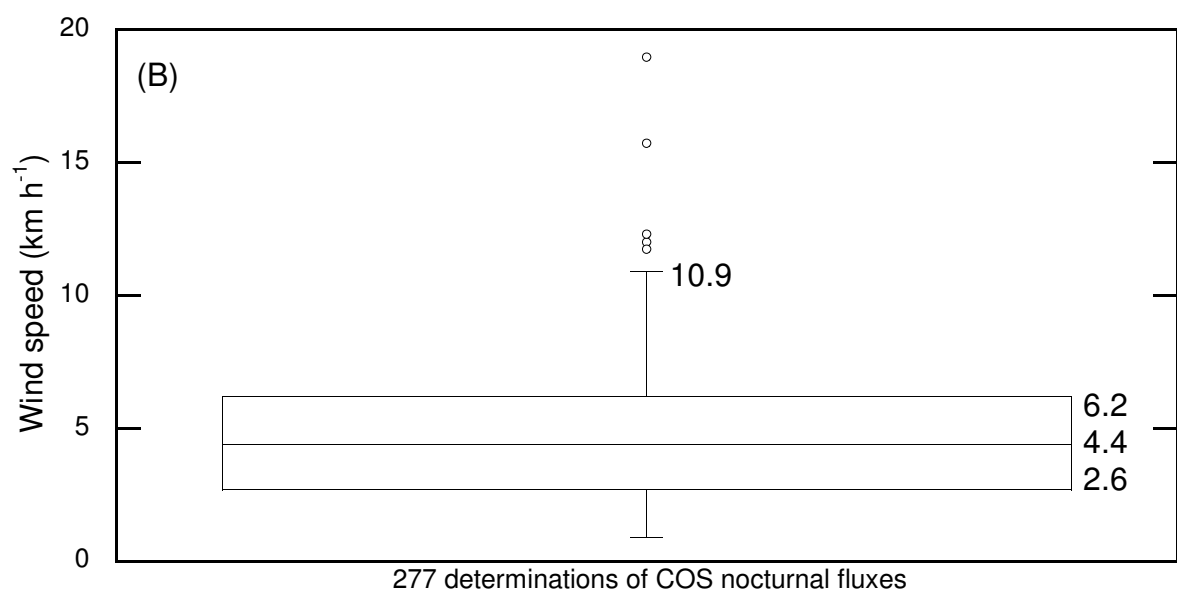

Supplement: S12 Fig — (A) Histogram and (B) basic statistics (median, upper and lower quartiles, outliers) of nocturnal (22h–4h UTC) wind speed (measured at 10 m height) corresponding to COS exchange rates calculated using the Radon Tracer Method (n = 277). (PDF) [file pone.0228419.s012.pdf]

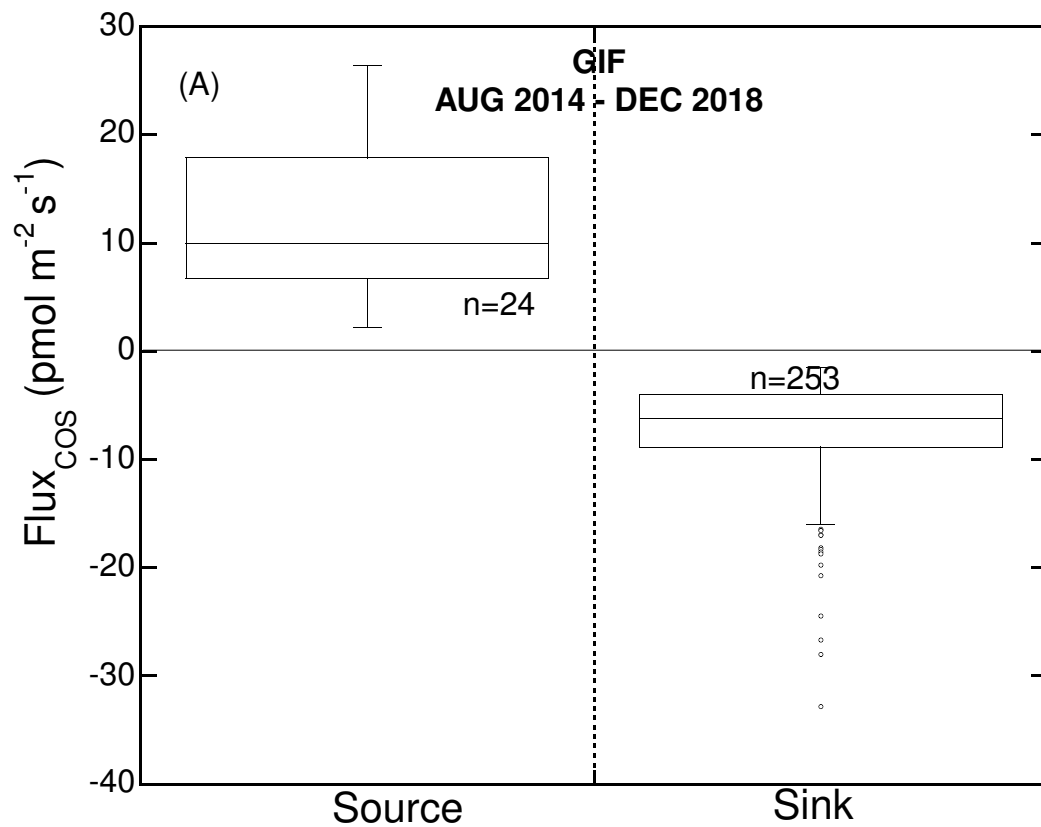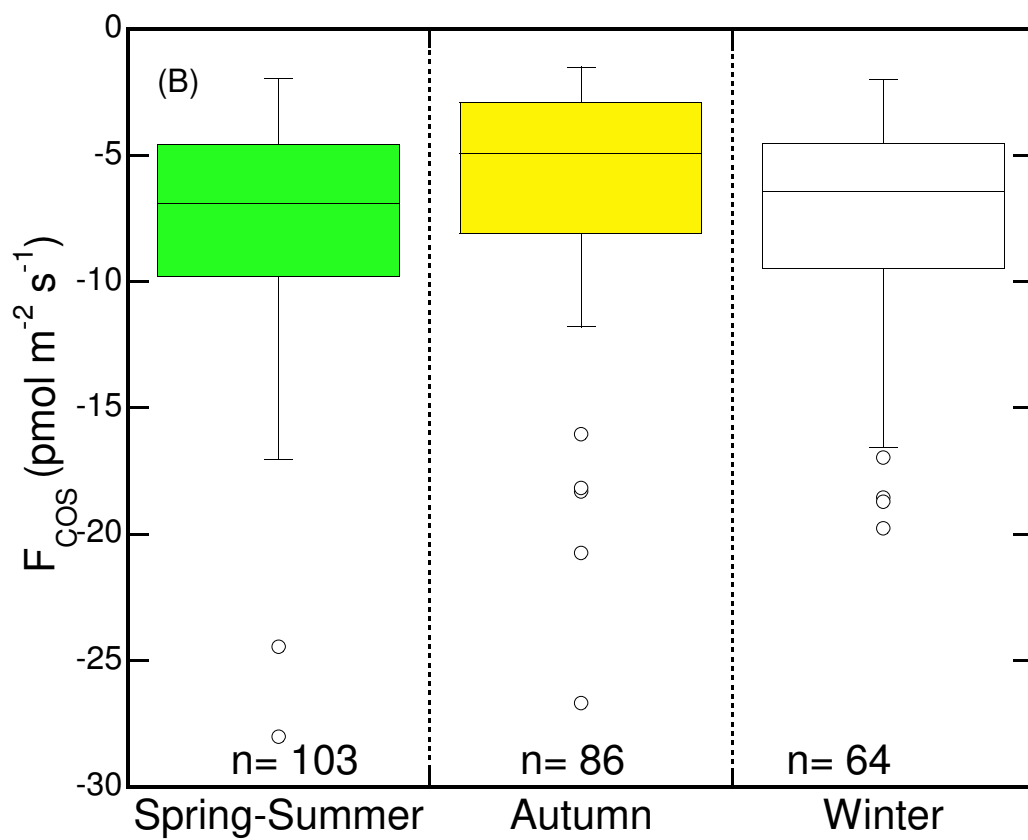

Supplement: S13 Fig — (A) Production and uptake rates. (B) Net uptake rates of COS sorted according roughly to seasons are color coded as follows: green (spring-summer), yellow (autumn) and white (winter). 10th, 25th, 50th, median, 75th and 90th percentiles were used. Circles correspond to outliers. Until radon data for 2019 are validated, here the period of concern extends from August 2014 to December 2018. (PDF) [file pone.0228419.s013.pdf]

NOAA HYSPLIT MODEL  
Backward trajectories ending at 1200 UTC 24 Jan 16  
GDAS Meteorological Data

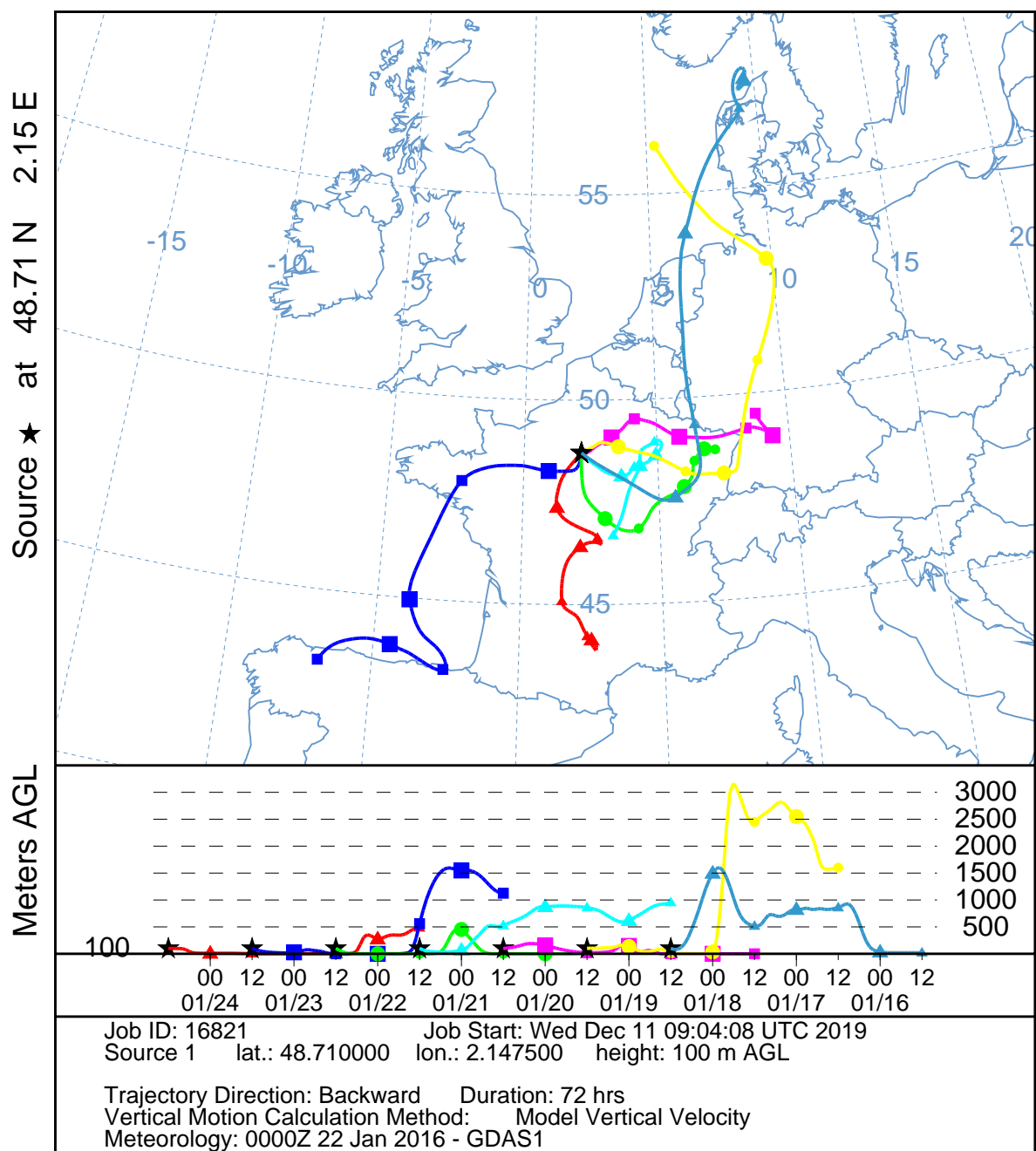

Supplement: S16 Fig — Computed at 12:00 UTC, 100 m agl, using HYSPLIT’s normal mode and GDAS1 meteorological data. Ending on 24 January 2016. (PDF) [file pone.0228419.s016.pdf]
