# Supplementary material for: A top-down approach of sources and non-photosynthetic sinks of carbonyl sulfide from atmospheric measurements over multiple years in the Paris region (France)
Source: PLoS One. 2020 Feb 10;15(2):e0228419. doi: 10.1371/journal.pone.0228419 (PMC7010246; doi:10.1371/journal.pone.0228419)
Supplement: S14 Fig — The period of concern is June 15 to July 15 when nocturnal Radon and COS enhancements were observed. Wind speed and direction were recorded at 10 m height. (PDF) [file pone.0228419.s014.pdf]

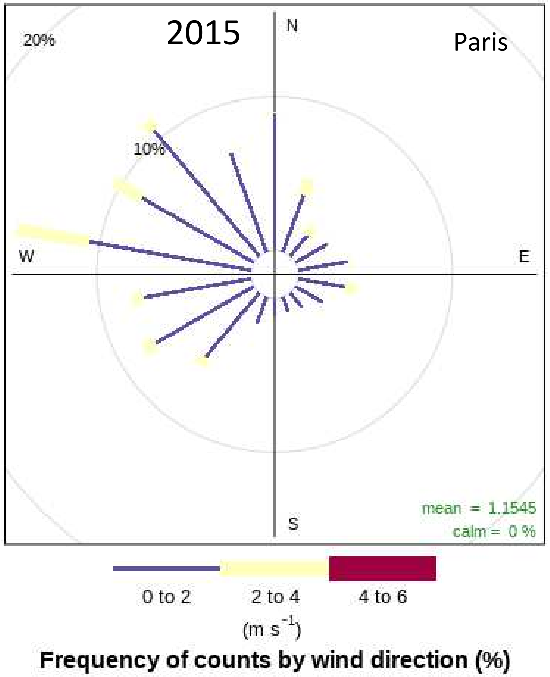
2015
Paris
N
S
W
E
20%
10%
mean = 1.1545
calm = 0 %
0 to 2
2 to 4
4 to 6
(m s-1)
Frequency of counts by wind direction (%)

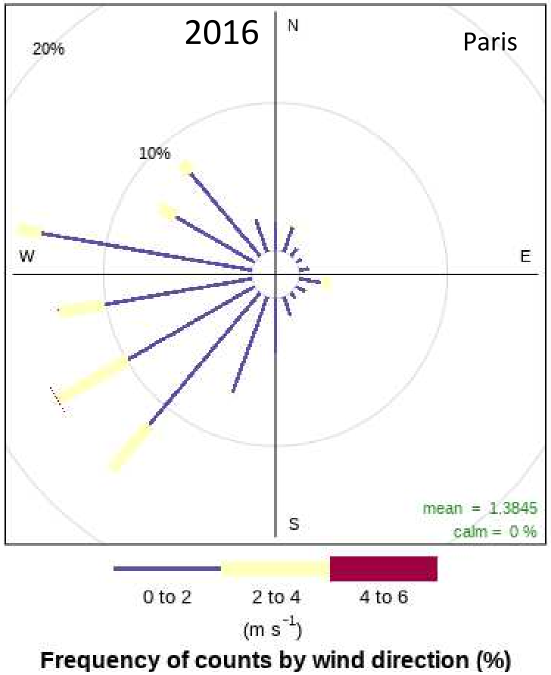
2016
Paris
N
S
W
E
20%
10%
mean = 1.3845
calm = 0 %
0 to 2
2 to 4
4 to 6
(m s-1)
Frequency of counts by wind direction (%)

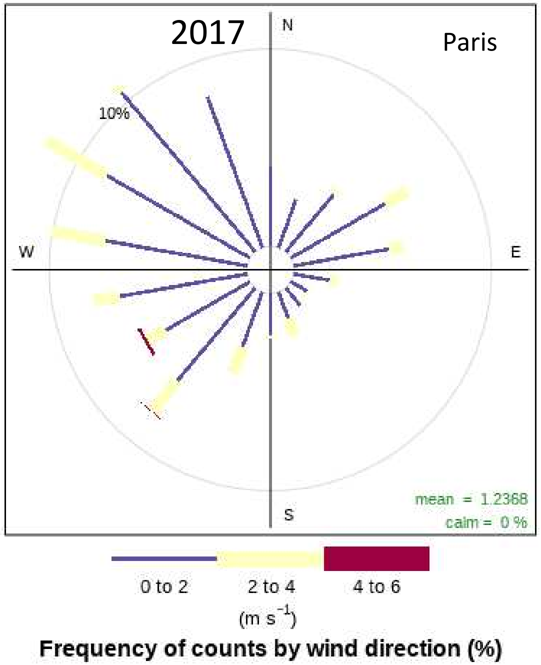
2017
Paris
N
S
W
E
10%
mean = 1.2368
calm = 0 %
0 to 2
2 to 4
4 to 6
(m s-1)
Frequency of counts by wind direction (%)

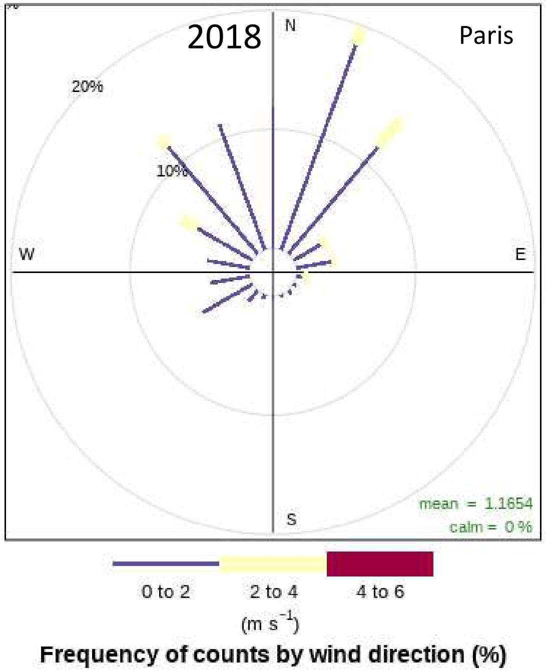
2018
Paris
N
S
W
E
20%
10%
mean = 1.1654
calm = 0 %
0 to 2
2 to 4
4 to 6
(m s-1)
Frequency of counts by wind direction (%)
