# Supplementary material for: A top-down approach of sources and non-photosynthetic sinks of carbonyl sulfide from atmospheric measurements over multiple years in the Paris region (France)
Source: PLoS One. 2020 Feb 10;15(2):e0228419. doi: 10.1371/journal.pone.0228419 (PMC7010246; doi:10.1371/journal.pone.0228419)
Supplement: S15 Fig — Computed at 12:00 UTC, 100 m agl, using HYSPLIT’s normal mode and GDAS1 meteorological data. Ending on (A) 14 February and (B) 22 March 2015. (PDF) [file pone.0228419.s015.pdf]

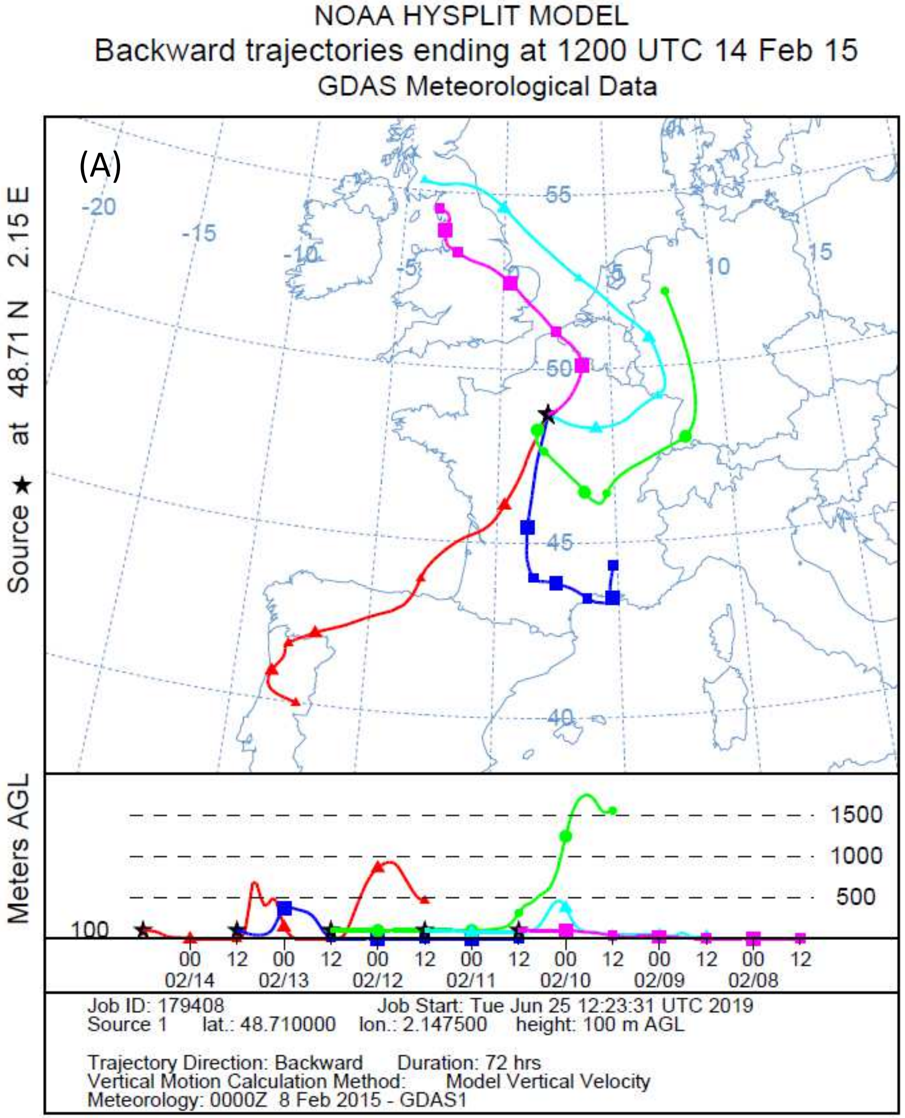
NOAA HYSPLIT MODEL
Backward trajectories ending at 1200 UTC 14 Feb 15
GDAS Meteorological Data
(A)
Source ★ at 48.71 N 2.15 E
Meters AGL
1500
1000
500
100
00 12 00 12 00 12 00 12 00 12 00 12 00 12
02/14 02/13 02/12 02/11 02/10 02/09 02/08
Job ID: 179408 Job Start: Tue Jun 25 12:23:31 UTC 2019
Source 1 lat.: 48.710000 lon.: 2.147500 height: 100 m AGL
Trajectory Direction: Backward Duration: 72 hrs
Vertical Motion Calculation Method: Model Vertical Velocity
Meteorology: 0000Z 8 Feb 2015 - GDAS1

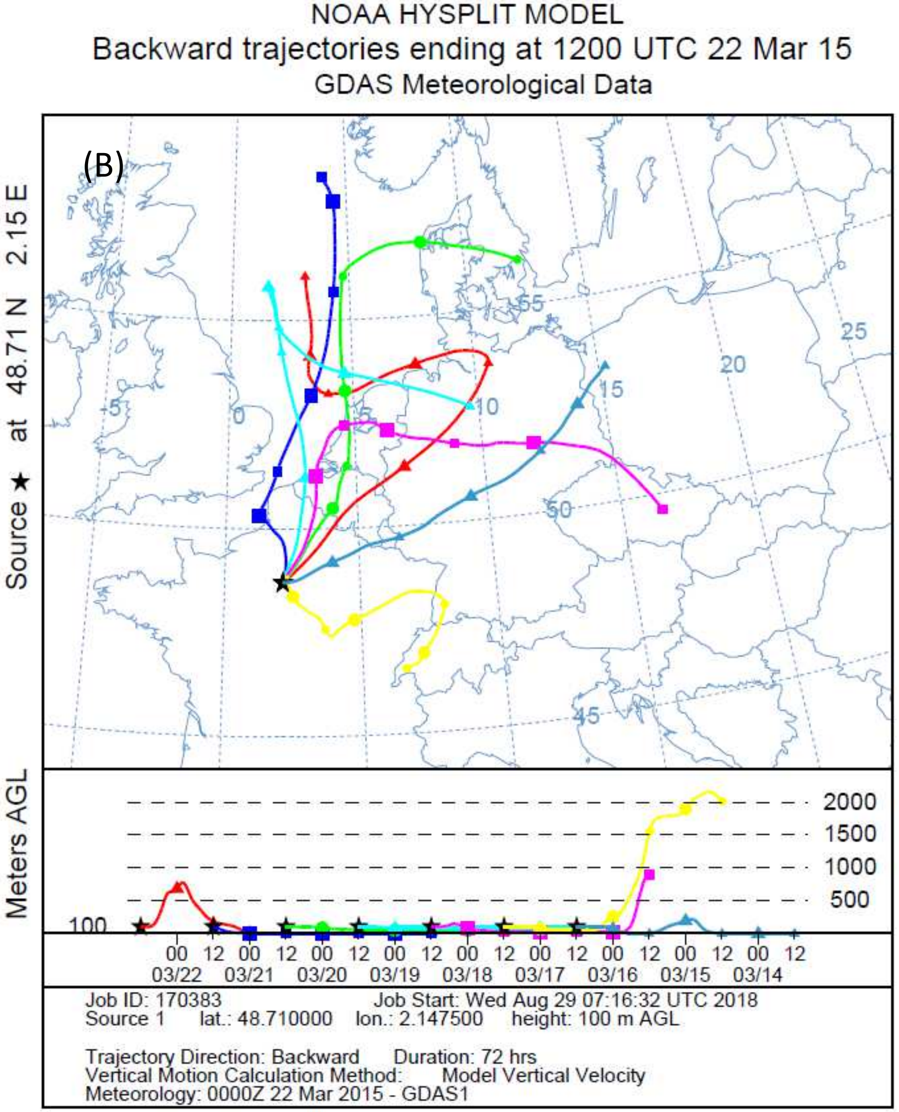
NOAA HYSPLIT MODEL
Backward trajectories ending at 1200 UTC 22 Mar 15
GDAS Meteorological Data
(B)
Source ★ at 48.71 N 2.15 E
Meters AGL
2000
1500
1000
500
100
00 12 00 12 00 12 00 12 00 12 00 12 00 12 00 12 00 12
03/22 03/21 03/20 03/19 03/18 03/17 03/16 03/15 03/14
Job ID: 170383 Job Start: Wed Aug 29 07:16:32 UTC 2018
Source 1 lat.: 48.710000 lon.: 2.147500 height: 100 m AGL
Trajectory Direction: Backward Duration: 72 hrs
Vertical Motion Calculation Method: Model Vertical Velocity
Meteorology: 0000Z 22 Mar 2015 - GDAS1
