# Supplementary material for: A top-down approach of sources and non-photosynthetic sinks of carbonyl sulfide from atmospheric measurements over multiple years in the Paris region (France)
Source: PLoS One. 2020 Feb 10;15(2):e0228419. doi: 10.1371/journal.pone.0228419 (PMC7010246; doi:10.1371/journal.pone.0228419)
Supplement: S17 Fig — Computed at 12:00 UTC, 100 m agl, using HYSPLIT’s normal mode and GDAS1 meteorological data. Ending on (A, B) 14 and 19 March 2016, respectively. (PDF) [file pone.0228419.s017.pdf]

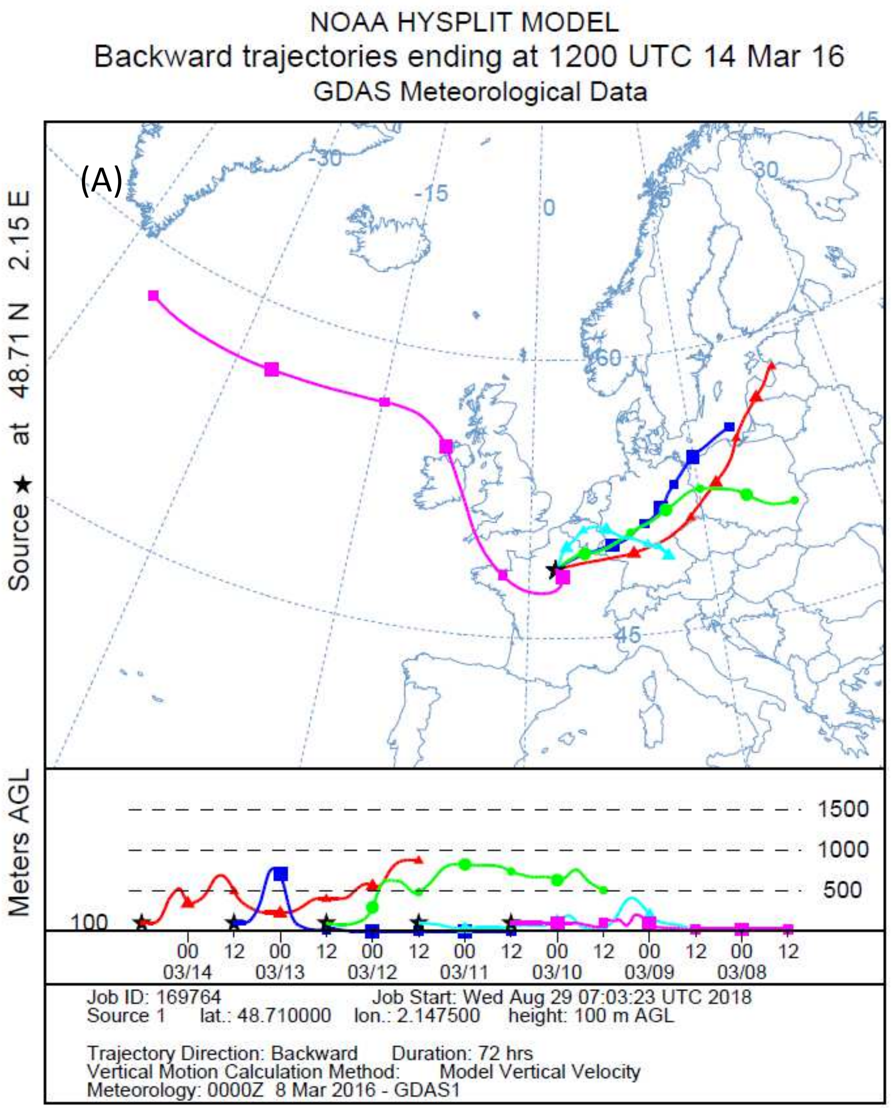

NOAA HYSPLIT MODEL
Backward trajectories ending at 1200 UTC 14 Mar 16
GDAS Meteorological Data
(A)
Source ★ at 48.71 N 2.15 E
Meters AGL
1500
1000
500
100
00 12 00 12 00 12 00 12 00 12 00 12 00 12
03/14 03/13 03/12 03/11 03/10 03/09 03/08
Job ID: 169764 Job Start: Wed Aug 29 07:03:23 UTC 2018
Source 1 lat.: 48.710000 lon.: 2.147500 height: 100 m AGL
Trajectory Direction: Backward Duration: 72 hrs
Vertical Motion Calculation Method: Model Vertical Velocity
Meteorology: 0000Z 8 Mar 2016 - GDAS1

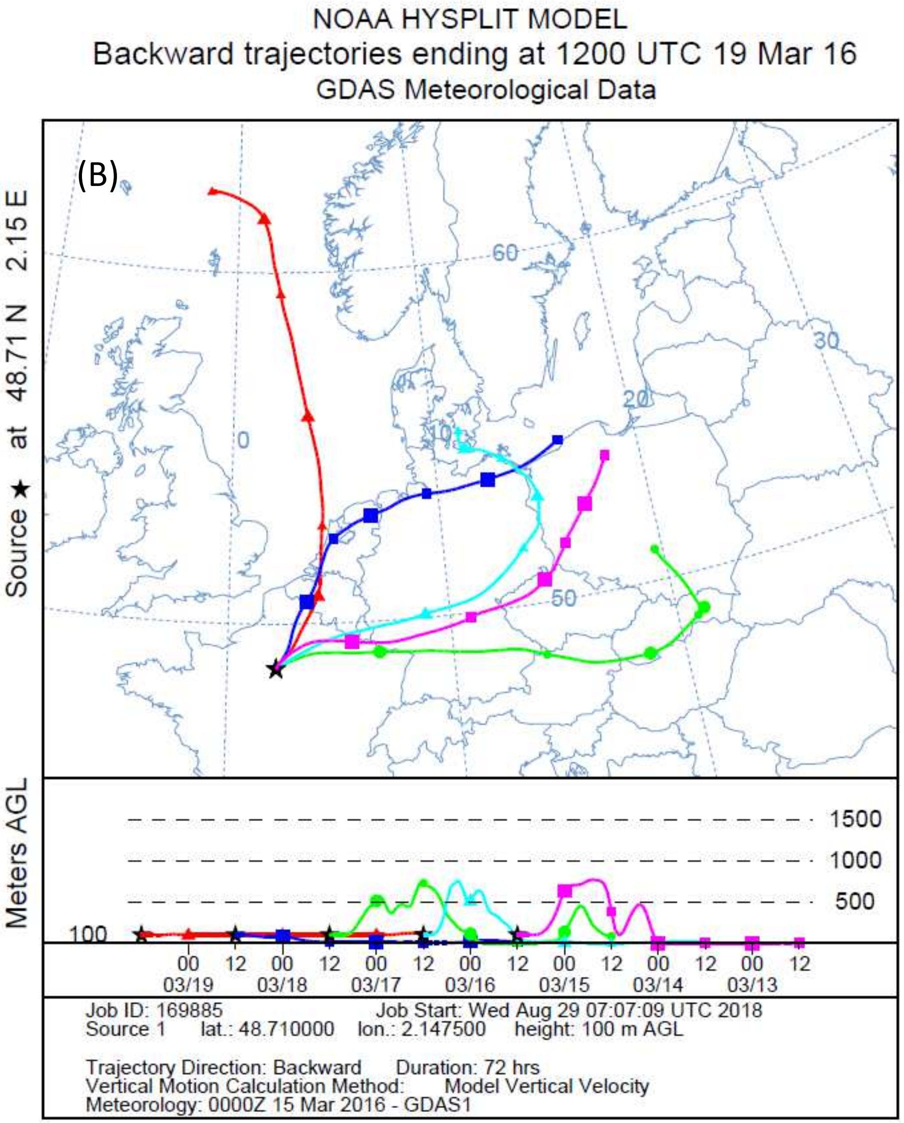

NOAA HYSPLIT MODEL
Backward trajectories ending at 1200 UTC 19 Mar 16
GDAS Meteorological Data
(B)
Source ★ at 48.71 N 2.15 E
Meters AGL
1500
1000
500
100
00 12 00 12 00 12 00 12 00 12 00 12 00 12
03/19 03/18 03/17 03/16 03/15 03/14 03/13
Job ID: 169885 Job Start: Wed Aug 29 07:07:09 UTC 2018
Source 1 lat.: 48.710000 lon.: 2.147500 height: 100 m AGL
Trajectory Direction: Backward Duration: 72 hrs
Vertical Motion Calculation Method: Model Vertical Velocity
Meteorology: 0000Z 15 Mar 2016 - GDAS1
