# Supplementary material for: A top-down approach of sources and non-photosynthetic sinks of carbonyl sulfide from atmospheric measurements over multiple years in the Paris region (France)
Source: PLoS One. 2020 Feb 10;15(2):e0228419. doi: 10.1371/journal.pone.0228419 (PMC7010246; doi:10.1371/journal.pone.0228419)
Supplement: Attachment — Submitted filename: Response to Reviewers.docx [file pone.0228419.s018.docx]

Academic editor appraisal **(in bold case)**, response to (in plain text)**:**

**The reviewers provided useful suggestions on improving the manuscript, especially the figures and figure captions. The method description should be amended according to the comments of Reviewer 1. We also recommend briefly discussing the issues that raised questions.**

In the following, we provide responses to each point raised by the academic editor and reviewers. Figures and their captions have been updated accordingly and former Figure 2 has been moved to supplementary material as suggested by reviewer 1.

Dr. C. Yver-Kwok, who is an expert in the assessment of fluxes using the Radon-Tracer Method (RTM) here at LSCE, authored/coauthored several articles cited in our manuscript where she provided a full description of how the fluxes are determined by RTM (e.g. Yver et al., 2009; doi:10.1029/2009JD012122) and explained how the local ^222^Radon exhalation rates were adjusted to the Gif-sur-Yvette site. For the equation how the fluxes are determined, quality screening and uncertainties related to the method, the reader is referred to pages 7 & 8 of Yver et al. (2009). Dr. Yver-Kwok also provided a code she wrote in IDL programming language that one of us (B. Lebegue) adapted for COS using the R programming language. To guaranty the conformity of our approach to previous ones, Dr. Yver-Kwok has agreed to be part of the list of co-authors as in Belviso et al. (2013; doi:10.3402/tellusb.v65i0.20719). Hence, the method description has been amended according to the comments of reviewer 1 only partially because it has already been published by the first author himself (Belviso et al., 2013) and co-authors (e.g. Yver et al., 2009).

In the revised manuscript, we have improved the discussion of issues that raised questions (e.g. the effect of boundary layer height to observations, guesses for the sources of COS during summer, fate of COS during transport of polluted air masses from the neighbouring countries to the sampling sites).

Reviewer evaluation **(in bold case)**, response to reviewer (in plain text)**:**

**Reviewer #1: The manuscript by Belviso et al. “A top-down approach of sources and non-photosynthetic sinks of carbonyl sulfide from atmospheric measurements over multiple years in the Paris region (France)” uses measurements of radon and atmospheric carbonyl sulfide (COS) concentrations from two different sites and few different vertical levels to determine sources and sinks of COS in the regional scale. The main finding is that due to undefined anthropogenic emissions and other undefined sources and sinks, the usage of COS measurements in determining photosynthesis is limited in the regional and global scales.

General comments:

Methods used in the study are sound but need to be clarified more in the text. Especially the “Methods” section needs to be revisited for better clarity and to help the reader. The main concern is about drawing the conclusions on emission and sink areas and processes affecting COS exchange and concentration based on the evidence showed in the manuscript. The authors should clarify the effect of boundary layer height to observations and conclusions. Also, the mechanism of how and why pollution events affect the atmospheric COS concentration remains unclear. Overall, this is an important and interesting study well suited for publication after some corrections, which are specified below.**

The “Methods” section has been revisited. Section entitled “COS and ^222^Radon measurements” is now divided in 3 subsections as follows:

“COS measurements by GC at the GIF site”

“Computation of COS fluxes with RTM at the GIF site”

“COS measurements by QCL at the SAC site”

Each subsection has been reorganized.

The effect of boundary layer height to observations is addressed using ^222^Rn measurements as follows:

In the “Methods” section it is now stated that: “^222^Radon, a tracer uniformly emitted by soils and whose variations near the surface are mainly caused by variations of planetary boundary layer height (PBLh) [17], was monitored at a height of 7 m at GIF. During nocturnal temperature inversions, the accumulation of ^222^Rn in the boundary layer results from a lowering of vertical mixing [10]. Hence, in the absence of PBLh estimates from lidar measurements, the PBLh effect to observations was assessed using ^222^Rn measurements.”

We have also clarified the mechanism of how and why pollution events affect the atmospheric COS concentration. During wintertime, COS enhancements in the ABL are observed only when PM pollution events originate from outside the Greater Paris Area especially when back trajectories of air masses are oriented from Benelux and Germany. When PM pollution events are restricted to the Greater Paris Area they are associated with losses of COS. Our observations are not consistent with the Greater Paris Area being a general source of COS as Zumkehr et al. (2018) suggest.

**Specific comments:

Methods: The method section should be more organized. Right now the reader gets a bit lost on when is which site or method discussed. The authors mention multiple times of calibration measurements but it is not clear whether these measurements are continuous and/or how frequent and at which site. Mention the main building/tree/vegetation height around the measurements for both sites. What is the measurement frequency of the QCL? How about averaging time? Tell more about the radon tracer method – show equation how the fluxes are determined and tell how the local exhalation rates were obtained. What is the footprint of this method and is there some quality screening and/or uncertainties related to the method? There should be a high correlation of Rn and COS concentrations for the method to work, did you check this? You could show in the supplement (correlation in addition to the time series of Fig. S1).**

The “Methods” section has been revisited as follows.

Section entitled “site description”

- At the local scale, the two sites are surrounded by roads and a highway, urban areas, croplands, grasslands, lawns (including a golf course between SAC and GIF) and forests to the west and south. Building and tree height does not exceed 7 m at the GIF site. Taller buildings exist at the SAC site but not in the immediate proximity of the sampling site.

Section entitled “COS and ^222^Radon measurements” is now divided in 3 subsections as follows:

“COS measurements by GC at the GIF site”

- Calibration was performed about every three weeks using a calibration gas commercialized by Air Products [16] and the drift between calibrations was assessed on a weekly basis using a short-term target gas.

“Computation of COS fluxes with RTM at the GIF site”

- ^222^Radon, a tracer uniformly emitted by soils whose variations near the surface are mainly caused by variations of atmospheric boundary layer height (PBLh) [17], was monitored at a height of 7 m at GIF. During nocturnal temperature inversions, the accumulation of ^222^Rn in the boundary layer results from a lowering of vertical mixing [10]. Hence, in the absence of PBLh estimates from lidar measurements, the PBLh effect to observations was assessed using ^222^Rn measurements. ^222^Radon was measured with the active deposit method, that is, via the radioactive decay of its daughters attached to aerosols, and was calibrated against an Australian Nuclear Science and Technology Organisation (ANSTO)-built detector for continuous monitoring of radon concentration in air [18]. Flux computations using the Radon-Tracer Method (RTM) are described in detail in [10-11]. In short, the RTM estimates nocturnal fluxes using (1) hourly COS mixing ratio observations, (2) time-averaged ^222^Rn values reported at the beginning of the two hour averaging interval, and (3) estimates of ^222^Rn local exhalation rates as by [11]. Fluxes are computed essentially as the product of COS/^222^Rn slopes and ^222^Rn local exhalation rates. Data that are used to calculate the slope of linear regression during night-time inversion (COS/^222^Rn) are selected according to the time of day as follows: [18; 6[ (UTC) in spring, [19; 5[ in summer, [17; 6[ in autumn and winter. In order to be considered significant, the nocturnal COS concentration increases or decreases and ^222^Rn concentration increases have to be greater than 15 ppt and 0.5 Bq m^-3^, respectively, and the coefficient of determination of the linear regression (r^2^) should be greater than 0.6. Two examples of fluxes, shown along with mixing ratio and concentration measurements and with r^2^ values, are displayed in S2 Fig for the nights of April 11, 2015 (negative flux, sink of COS) and July 9, 2016 (positive flux, source of COS).

“COS measurements by QCL at the SAC site”

- The measurement frequency of the QCL is 1 Hz.

- CAL, dry gas analyzed about every fifteen hours

**Results: How is the soil composition affecting the results? How about boundary layer height? How much measurement uncertainties affect the results?**

In the last paragraph of section entitled “Multi-year variations of COS mixing ratio and exchange rates”, we evaluate the seasonality of emission fluxes in the GIF area in the light of our current understanding of emission processes. As far as we are aware of, the effects of soil temperature, soil water content, incoming solar radiation and crop residues on emission fluxes are better understood than those of soil composition although the latter is receiving increasing attention in aspects concerning the enzymatic activity and microbiology of soils (e.g. Meredith et al., 2019, doi.org/10.1038/s41396-018-0270-2). Moreover, within the distance of 4.5 km from the monitoring station, several soil types with very contrasted compositions are observed as described in the material and method section. It thus seems more reasonable to discuss the effect of environmental conditions (temperature, humidity, incoming solar radiation) relatively constant over the footprint area than the effect of soil composition that is very variable. In the revised version, it is now suggested that rapeseed crops rather than processes taking place in the soil would be responsible for the recurrent early summer emissions of COS in the GIF area. For boundary height issues see above (The effect of boundary layer height to observations is addressed using ^222^Rn measurements as follows…). The uncertainties on the COS fluxes come from the regression COS/Rn (see the revised version of Fig 3). **Fig 1: Add the scales of the maps. Consider changing colors of the site abbreviations in the map, not clearly visible in 1B. Three shades of pink were not distinguishable on computer screen and texts not distinguishable in pdf or printed versions. From 1A you get the expression that only GIF site is there, consider making some adjustments.**

This figure has been revised.

We added latitudes and longitudes and the scale of the maps, but the colors and the site abbreviations can’t be changed because these are the “official” ones designed by CESBIO from the online application at <https://www.theia-land.fr/en/product/land-cover-map/>. The resolution is the best achievable. For example, in the upper left corner of the right panel (B), one can notice the presence of the two landing runways of a small airfield (Toussus-le-Noble). Moreover, it is possible to follow the track of highway N118 from the upper right corner down to the lower right corner.

**Fig 2: Unclear caption. How often was the cylinder measured? Could be moved to supplementary material with other calibration/LTR/STR figures.**

The cylinder was measured about every three weeks. Former Figure 2 has been moved to supplementary material as suggested by the reviewer. **Fig 3: Why not use the same x-axis ticks in the different subplots? Caption for this is confusing. Check the months mentioned and also list which months are marked with white. Why are they white and not part of different seasons? Transition periods?**

This figure has been revised accordingly. Transition periods are shown in white. **Fig 4: Are you sure the high positive and negative fluxes are not outliers or bad quality? Mention in the caption that these are nocturnal fluxes obtained by radon tracer method.**

All data points plotted in Fig. 3 passed the stringent selection procedure described in the subsection entitled “Computation of COS fluxes with RTM at the GIF site”. So the high positive and negative fluxes are not outliers or bad quality but, according to error bars, display higher uncertainty.

**Fig 5: Mention which year are the measurements.**

Done**.

Fig 6: Proper legends needed, now the reader gets confused by lines and dots not explained in the legend. Also the caption is a bit misleading as the colors are not properly explained. The two COS signals seem to be quite similar to each other: what are the occasions when they differ and why?**

This figure has been redrawn partially and the legend modified as follows: “Hourly variations in ^222^Rn activity and COS mixing ratio at the GIF and SAC sites. (A) For December 2015 – January 2016 and (B) March 2016. The blue and black lines correspond to COS data at SAC (100 m) and ^222^Rn data at GIF (7 m), respectively. SAC data at 15 m are not shown because there was no significant vertical gradient between 15 m and 100 m. The red line in panel A and the red dots in panel B correspond to COS data at GIF. The red line in panel B corresponds to COS data at SAC (15 m). The yellow bands correspond to PM pollution episodes over the “Informing” or the “Alert” thresholds defined by AIRPARIF.”

Reviewer is right because the two sites are located at short distance (2 km) of each other and because there was no COS vertical gradient between 7 m and 100 m at the GIF/SAC sites even during nighttime (same for CO_2_). In other words, the inlet height at 100 m remained within the nocturnal boundary layer from early December 2015 to late January 2016. That is why we could fill the gaps at 7 m with data collected at 100 m and *vice versa*. The two signals differ when the tower height is higher than the nocturnal boundary layer. This rarely happened at the SAC site during the six months survey of COS with QCL.  **Fig 7: What does the unit mean? Mention in the caption that these are during the pollution events.**

Sorry for the mistake. The unit is s.m^-1^ not s.m^-2^. When footprints expressed in s.m^-1^ and fluxes in g.m^-2^.s^-1^ are combined, the product is a concentration in g.m^-3^. In other words, by multiplying a footprint map by a flux map, one can evaluate the contribution of fluxes along the trajectory of an air mass to concentrations measured at the point of observation (e.g. at https://www.atmos-chem-phys.net/4/51/2004/).
Done as suggested.

**Fig S1: Explain what is the grey band.**

The night length explains why the grey band is narrower in summer than in spring. **Fig S4&S5: Is this at the SAC site?**

No, we used a cylinder of target gas in the laboratory not in the field. **Fig S8: Use the same scales in x and y axis for clarity**

Done. **Fig S9: How many analyses the three points consist of?**

The duration of QCL analyses and the number of GC determinations of COS mixing ratios are now displayed in the revised figure. **Fig S11: A histogram would be more representative**

Done. **L22: suggest to replace “eddy flux scale” with “ecosystem scale”, also further on in the manuscript**

Done.

**L67-70: Mention that this applies to the regional scale**

Done.

**L84-87: Complicated sentence, simplify for easier read**

It has been rewritten as follows:

“The Greater Paris Area occasionally faces intense pollution episodes during stable atmospheric conditions notably during winter (e.g. [14]). COS and ^222^Radon measurements were also used to assess the significance of Greater Paris Area anthropogenic emissions.”

**L103: Mention which scale you are talking, seems like the sites are mostly surrounded by urban areas and croplands, while forests do not seem to be dominating**

We talk about the local scale.

**L124: How are the thresholds defined and what difference between “A” and “I”?**

The sentence has been modified as follows:

“The definitions of thresholds can be found in a table named “Tableau des seuils” by clicking on the above URL. For PM_10_, “A” is reached when average daily concentrations exceed 80 µg m^-3^ (50 µg m^-3^ in the case of an episode labelled “I”).”

**L129: Which GC and for which gases, is this an automated sampling system?**

The sentences have been modified as follows:

“At the GIF site, all sampling lines were made of Synflex tubing which is suitable for COS analysis [13]. Air was sampled 7 m above ground level on the top of a building for about four and a half years, and analyzed on an hourly basis by gas chromatography (GC). The COS analytical measurement technique is described in [16]. In short, COS is trapped and desorbed using an automated sampling system (Entech P7100 preconcentrator), then analyzed by gas chromatography with pulsed flame photometry detection (PFPD, Varian Model 3800 [17]).”

**L147: What was calibrated? Complicated sentence.**

Former Fig 2 is now presented in the supplement S1 Fig and the legend has been slightly modified as follows:

“Accuracy and long-term repeatability over 4.5 years for atmospheric COS. The air compressed cylinder, prepared and certified by NOAA-ESRL, was analyzed by gas chromatography with PFPD detection. The GC was calibrated using a calibration gas purchased from Air Products. The LTR here expressed as the SD of the average of 96 determinations over 4.5 years is 8.6 ppt. From October 2018 to March 2019 the cylinder was not used to increase its service life.”

**L159: Did you follow NOAA-ESRL recommendation and use stainless steel cylinders?**

NOAA-ESRL recommendation to use stainless steel cylinders has been released recently. We will purchase stainless steel cylinders from NOAA in the future.

**L172-175: These seem more like results rather than methods**

The sentence has been rewritten as follows:

“Two examples of fluxes, shown along with mixing ratio and concentration measurements and with r^2^ values, are displayed in S2 Fig for the nights of April 11, 2015 (negative flux, sink of COS) and July 9, 2016 (positive flux, source of COS).”

**L177: “performances” refering to multiple QCL?**

We now use “specifications” instead of “performances”. “Before being deployed at the SAC site, the specifications of our mini QCL instrument were evaluated in…”

**L199: This is repetition from L194**

We modified the sentence as follows:

“In that case, the instrument did manage rather well the fluctuations in laser light intensity.”

**L205: Why this large temperature variation? In what kind of room is the QCL placed?**

Temperature variations are applied by increasing heating in the room.

**L205-208: Not clear whether there was a temperature dependence or not**

The coefficient of determination (r^2^) of the linear regression is equal to 0.02. So, there is no temperature dependence in the sense that it is not possible to use an equation to correct COS mixing ratios for cell temperature changes.

**L219: Which unit is [COS]?**

PPT.

**L317: Do you mean that the small difference in the COS sink between seasons is unlikely observed over forests, that would have a larger difference? Currently this is not very clear.**

In some forests it has been shown from eddy covariance measurements and soil enclosures that the main contribution to the observed COS drawdown at night is from plants. Our observations suggest that the COS drawdown at night in the GIF area is not due to plants but to soil processes.

**L331-340: Where do you show evidence on solar radiation, temperature or dry conditions effect on COS exchange?**

In temperate areas as in GIF, hotter and drier conditions are observed during summer and irradiance is higher. Evidences of solar radiation, temperature or dry conditions effect on COS exchange have been provided in articles published by others. References are cited in the text. Sorry if we did not catch the point you raise.

**L358: From Fig. 5 it seems more that COS signal is lagging Rn signal rather than being in phase. Please check this carefully from the data. In case of a phase difference exists, how long is it?**

We think that this is logical because when remote transport of COS anthropogenic emissions takes place, the COS signal can lag ^222^Rn signal rather than being in phase (Figs 4B, 5B) because COS can be removed during transport by biogenic sinks at ground level and ^222^Rn accumulate concomitantly. The existence of a phase difference between COS and ^222^Rn signals prevents these events to be detected and quantified as local emission events using the RTM. In other words, COS enhancements in the ABL are observed only when PM pollution events originate from outside the Greater Paris Area especially when back trajectories of air masses are oriented from Benelux and Germany, but this does not necessary require ^222^Rn and COS signals to be strongly correlated as at the local scale during nighttime inversions.

**L368: Could this be partly due to precision of measurements?**

Precision at 15 min averaging time is about 5 ppt (S3 Fig). Hence, a vertical gradient of a few tens of ppt (30 ppt at the most) observed occasionally in December 2015 is significant.

**L377-380: A confusing sentence. Elsewhere?**

“…will be addressed in a manuscript in preparation.” is indeed more appropriate.

**L382-388: I believe this is mainly due to boundary layer height? In stable conditions COS is taken up by plants while emissions coming from elsewhere cannot penetrate to the shallow stable boundary layer, and thus the concentration goes down. So I am not sure you can say “anthropogenic emissions of COS are offset by natural uptake” as it is just a question of where and what height the observations are done.**

This is not correct because in that case (February 2015) the COS anthropogenic emissions are not coming from elsewhere but from the Greater Paris Area itself (Zumkehr et al., 2018). This is a strong indication that the anthropogenic emissions of COS are offset by natural uptake in the Paris area because the juxtaposition of anthropogenic sources and natural sinks at ground level in the Greater Paris Area allow fluxes to be transported and mixed laterally within the planetary boundary layer during inversion episodes of several day duration. We have clarified this point in the revised version and provide below a map of Zumkehr’s inventory showing that our sampling area is supposed to be a strong (≥ 100 pmol m^-2^ s^-1^) source of anthropogenic COS.

 **L389: Write what is the emission reported by Zumkehr et al. [9]**

The emission reported by Zumkehr et al. (2018) is 98 pmol m^-2^ s^-1^ in the GIF area as shown above (red triangle inside a yellow square).

**L392-394: Not sure you can draw this conclusion based on Fig. 7. The resolution is not great and the different subplots differ from each other quite a lot.**

Only subplot 6A (former Fig 7A) differs from the other subplots. When air masses are oriented from the SO the ABL loses COS over the Greater Paris Area. When air masses are oriented from the E-NE the ABL gains COS of anthropogenic origin from outside (Fig 6B-D).

**L469: Do you have any guesses for the sources?**
We believe that the role of agricultural fields should be investigated because some crops like oilseed rape plants, which are grown in the GIF area, have recently been shown to release COS at least in certain conditions as in response to fungal infection [35].The conclusion is unchanged but the discussion has been updated accordingly.

Reviewer evaluation **(in bold case)**, response to reviewer (in plain text)**:**

**Reviewer #2: The Manuscript by Belviso et al. brings out quality COS data over several years in France. Apart from its importance to the sulphate budget and stratospheric aerosols, COS measurements have found renewed application in estimating the C cycle. Despite the potential of COS measurements, quality measurements around the globe characterising different sources and ecosystems are still lacking, making budget estimations difficult. In this scenario, this work is not only interesting (and sound) in its findings of different source influences but also very important from the data point of view. I recommend publication of this paper in Plos One.

Some suggestions:
Figure 1: needs to be of much better resolution, with scales, latitude, and longitude marked for the global audience. Probably modify it to show the location in a global context first (see Fig 1 of https://doi.org/10.1016/j.scitotenv.2016.02.014 for an example)**

Figure 1 has been revised partially as shown below.

We added latitudes and longitudes and the scale of the maps, but the colors and the site abbreviations can’t be changed because these are the “official” ones designed by CESBIO from the online application at <https://www.theia-land.fr/en/product/land-cover-map/>. The resolution is the best achievable. For example, in the upper left corner of the right panel, one can notice the presence of the two landing runways of a small airfield (Toussus-le-Noble). Moreover, it is possible to follow the track of highway N118 from the upper right corner down to the lower-right corner of panel B. **Section: COS and Rn measurements
How do you ensure that there is no contribution due to in-situ atmospheric chemistry (e.g. oxidative addition of RSCs) during or after sampling or in sample line?**

The chemical lifetime of tropospheric COS is of several tens of years (Brühl et al., 2012). It is well established that COS is much less reactive and water soluble than gases like SO_2_ and H_2_S for which the use of oxidant (OH radical, O_3_) scrubbers is required before cryogenic trapping (Mallik et al. 2016 and references therein). Air samples being dehumidified through a Silonite coated SS trap at -25°C before cryogenic trapping, we do not expect COS hydrolysis to take place during desorption and transfer into the capillary column. All internal lines of the Entech preconcentrator are Silonite coated.

Our sampling line is made of about 50 meters of one-half inch OD Synflex tubing (total internal volume of about 3.2 liters). Being flushed permanently at a flow rate of about 6 L min^-1^, it is highly unlikely that COS could deposit or be oxidized in the sample line during a transit time of less than one minute. Moreover, Kooijmans et al. (2016) tested the interaction of Synflex with COS: no significant differences in COS mole fractions were found when the air flow was passed over Synflex (0.7±2.6 ppt). Synflex has also been used by Berkelhammer et al. (2014) for atmospheric COS sampling.

Berkelhammer, M., Asaf, D., Still, C., Montzka, S., Noone, D., Gupta, M., et al. (2014). Constraining surface carbon fluxes using in situ measurements of carbonyl sulfide and carbon dioxide. Global Biogeochemical Cycles, 28, 161–179. doi: 10.1002/2013GB004644.

Brühl, C., Lelieveld, J., Crutzen, P. J., and Tost, H. (2012). The role of carbonyl sulphide as a source of stratospheric sulphate aerosol and its impact on climate. Atmos. Chem. Phys., 12, 1239-1253. doi: 10.5194/acp-12-1239-2012.

Kooijmans, L. M. J., Uitslag, N. A. M., Zahniser, M. S., Nelson, D. D., Montzka, S. A., and Chen, H. (2016). Continuous and high precision atmospheric concentration measurements of COS, CO2, CO and H2O using a quantum cascade laser spectrometer (QCLS). Atmos. Meas. Tech., 9, 5293–5314. doi:10.5194/amt-9-5293-2016.

Mallik C, N., Chandra, S. Venkataramani, and S. Lal (2016). Variability of atmospheric carbonyl sulfide at a semi-arid urban site in western India. Sci. Total Environ., 551-552, 725-737. doi: 10.1016/j.scitotenv.2016.02.014.

**Lines 226-231: it would be good to show this as a supplementary figure or by just plotting the two instruments along the same axis.**

This was shown in former S8 Fig now numbered S9 Fig. Panels A and B of S9 Fig are now quoted in the revised paragraph. **Figure 3: Looking closely, there is also a decreasing tendency in COS around early June every year. What would be a possible cause? Would it be possible to look into relationships with soil temperature, PAR etc. (you can have a look at https://doi.org/10.1016/j.scitotenv.2016.02.014 for an example)?**

You are right. The decreasing tendency in COS around early June every year is a normal trend because the vegetation actively takes up atmospheric COS. It is the rising tendency in late June early July which is abnormal because the ecosystem shifts from a COS sink to a source.

In a previous version of the manuscript, COS fluxes and soil temperatures were displayed in a same plot, reproduced below.

Figure shows year-round estimates of COS fluxes (black dots) during nocturnal stable conditions (Gif-sur-Yvette, August 2014 to July 2017). Soil temperature (red line) is recorded about 50 cm below the surface at a weather station located 2 km North of the GIF sampling station. The uptake of COS (negative fluxes) appears to be insensitive to variations in soil temperature. The late spring and early summer COS production is indeed simultaneous with soil temperatures reaching 15°C to 20°C at 50 cm-depth in June and July and consequently surface soil temperature likely higher than the 15°C identified by Maseyk et al. (2014) as the transition of soil from a sink to a source of COS. However, COS production stopped at least one month before soil temperature began to decrease. COS production was moreover still observed in late autumn when soil temperature dropped at 50 cm-depth below 15°C, i.e. surface soil temperature considerably lower, and should favor COS uptake (Maseyk et al., 2014). Hence, data suggest that COS production is insensitive to variations in soil temperature too. Failure of the temperature sensor in 2018 as in 2015 drove us to the conclusion that the record of soil temperature was not of sufficient quality to be published in PLOS ONE.

Maseyk, K., Berry, J. A., Billesbach, D., Campbell, J. E., Torn, M. S., Zahniser, M., & Seibt, U. (2014). Sources and sinks of carbonyl sulfide in an agricultural field in the Southern Great Plains, Proceedings of the National Academy of Sciences, 111, 9064–9069. https://doi.org/10.1073/pnas.1319132111.
